# DNA Template Dependent Accuracy Variation of Nucleotide Selection in Transcription

**DOI:** 10.1371/journal.pone.0119588

**Published:** 2015-03-23

**Authors:** Harriet Mellenius, Måns Ehrenberg

**Affiliations:** Department of Cell and Molecular Biology, Biomedical Center, Uppsala University, Box 596, 751 24, Uppsala, Sweden; Hong Kong University of Science and Technology, HONG KONG

## Abstract

It has been commonly assumed that the effect of erroneous transcription of DNA genes into messenger RNAs on peptide sequence errors are masked by much more frequent errors of mRNA translation to protein. We present a theoretical model of transcriptional accuracy. It uses experimentally estimated standard free energies of double-stranded DNA and RNA/DNA hybrids and predicts a DNA template dependent transcriptional accuracy variation spanning several orders of magnitude. The model also identifies high-error as well a high-accuracy transcription motifs. The source of the large accuracy span is the context dependent variation of the stacking free energy of pairs of correct and incorrect base pairs in the ever moving transcription bubble. Our model predictions have direct experimental support from recent single molecule based identifications of transcriptional errors in the C. elegans transcriptome. Our conclusions challenge the general view that amino acid substitution errors in proteins are mainly caused by translational errors. It suggests instead that transcriptional error hotspots are the dominating source of peptide sequence errors in some DNA template contexts, while mRNA translation is the major cause of protein errors in other contexts.

## Introduction

Accurate transmission of sequence information in DNA to functional RNA molecules and proteins is essential for life. Transmission mistakes due to nucleotide substitution errors in transcription lead to dysfunctional RNA molecules and dysfunctional proteins. Gene transcription and aminoacylation of tRNA, with error frequencies in the 10^-5^ to 10^-4^ range [1; 2], have traditionally been considered as more accurate than translation, with an average error frequency of 10^-4^ [1], but *in vivo* estimates of the frequency of specific amino acid substitution [3] or transcription errors [2] have been scarce. It has in the past been difficult to distinguish authentic transcriptional errors from abundant sequencing and reverse transcription errors. However, in two recent studies an overall transcriptional error rate was estimated to 10^-5^ in *Escherichia coli* [2] and, using a single RNA molecule based experimental design, to 10^-6^ in *Caenorhabditis elegans* [4], marking a major breakthrough in the study of transcriptional accuracy.

Recent biochemical data on selection of aminoacyl-tRNA by the mRNA programmed ribosome suggest large variation of translation errors in the living cell [3]. In contrast to the previous assumption that translation errors effectively mask the influence of aminoacylation and transcription errors [5; 6], these novel observations suggest that translation errors may dominate in some, but not all, contexts. This conclusion is reinforced by the large, DNA context dependent accuracy variation about the mean predicted in the present work for nucleotide selection by transcribing RNA polymerase.

Here we have used computational modeling to analyze how variation in the standard free energy of the transcription bubble affects transcriptional errors in a DNA context dependent manner. We take advantage of a pioneering study by Yager and von Hippel, which shows how the free energy of the transcription bubble can be represented by the free energies of base pair formation and melting during each nucleotide incorporation cycle [7]. Our model combines the Yager and von Hippel theory with Eyring’s transition state approach to estimate rate constants for transitions between the different states of the transcription bubble [8]. As input data for the computational modeling of the template context dependent kinetics of incorporation of cognate and non-cognate nucleotides, we use the extensive and accurate experimental datasets that now exist on melting and formation free energies of DNA/DNA [9] and DNA/RNA [10] base pairs. Following the principle of Occam’s razor, we assume that the RNA polymerase enhances the accuracy of nucleotide selection in a context independent manner, e.g. by providing stereospecificity to cognate base pair recognition similar to the tRNA recognition by the translating ribosome [11]. Accordingly, our approach allows estimation of the DNA template dependent accuracy *variation*, but not the *absolute* RNA polymerase dependent accuracy level. Even in this simplified form, main features of our accuracy model have experimental support from recent data on transcriptional errors in the transcriptome of *C*. *elegans* [4]. Although sparse, this data set is particularly significant since it was created by a single molecule approach for error identification which ensures unambiguous separation of authentic transcription errors from artifacts due to other sources like reverse transcription or PCR.

**Fig 1:**
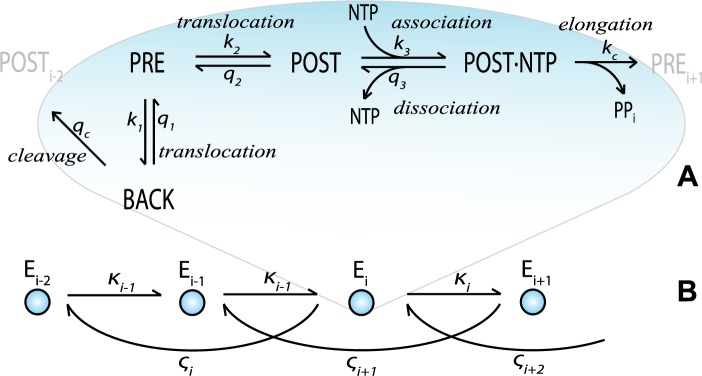
Reaction schemes of transcript elongation. A) Reaction scheme for the four states of the nucleotide addition cycle. B) Reaction scheme for the elongation states, determined by the transcript and the compounded rate constants that connect them: the κ-parameters signify forward movement by phosphodiester bond formation and the ς-parameters backtracking and dinucleotide cleavage of the nascent transcript. Note that the compounded rate constants in Fig. 1B are defined by the detailed rate constants in Fig. 1A, as described (see Methods for details).

Our results propose that the accuracy of initial nucleotide selection for phosphodiester bond formation splits into a discrete spectrum, determined by the base stacking of cognate and non-cognate base pairs against the previously incorporated base (Fig. 1). In contrast, the error spectrum for removal of an already incorporated base by proofreading appears to be almost continuous. This difference between the two selection spectra reflects the much larger kinetic complexity of the proofreading in relation to the initial selection mechanism (Fig. 1). The modeling further suggests that transcriptional errors involving the same nucleotide mismatches may vary by several orders of magnitude with the different sequence contexts of the DNA template. Our approach also reveals the existence of a context dependent enhancement of the accuracy of transcription of ribosomal RNA operons in *E*. *coli*, suggesting an evolutionary pressure on DNA template contexts for transcriptional error reduction. We predict the existence of hotspots for very high as well as very low errors, where the latter have inefficient incorporation also of cognate nucleotides due to the universal trade-off between rate and accuracy in enzymatic reactions [6; 12; 13].

## Results

### The model

We describe RNA polymerase movement along the DNA template during transcript elongation as a random walk between states of different standard free energies [14], with the current distance to the site of transcript initiation defined by the length of the nascent transcript (Fig. 1). The transcription bubble consists of 12 base pairs (bp) of unwound DNA and an 8 or 9 bp DNA/RNA hybrid that, together with the RNA polymerase, form the ternary elongation complex (TEC) [15; 16]. For each template position, the TEC may either translocate forward, to clear the catalytic site for binding of the next incoming nucleotide, or translocate backward, allowing for transcript cleavage and proofreading [17; 18]. The TEC is thermodynamically driven in the forward direction [19] by the ensuing chemical potential decrease due to RNA chain elongation and pyrophosphate release. These reactions and the four states of the transcription complex; pre-translocated (PRE), post-translocated (POST), back-translocated (BACK) and nucleotide associated (POST·NTP) complex; are illustrated in Fig. 1A.

The standard free energy of the transcription bubble is represented by a sum of free energies that changes through the transcription process by base pair formation and melting [7]. The reaction rate constants are modeled with the help of Eyring’s transition state theory [8]. In line with biochemical [20] and structural [21] data, backtracking is in our model confined to one step (Fig. 1A), and therefore a dinucleotide is discarded in every proofreading event [22; 23].

The rate constants connecting any two states of the nucleotide addition cycle are calculated by taking their standard free energy difference, as specified by the transcription bubble, into account. The standard free energy of any DNA/DNA [9] or DNA/RNA [10] double helix follows from the experimentally estimated nearest-neighbor parameters for every pair of base pairs, which predict the free energy change by the reaction (see Methods). The stability of each pair of base pairs depends strongly on the base stacking between them, which results in a large sequence dependent variation in free energy. We derive the rate constants of each reaction in the model by the introduction of a free energy barrier between initial and final reaction states, as in earlier kinetic models for transcription [8; 24]. We note that the accuracy of substrate selection is mainly determined by ratios of rate constants (see below), which reduces modeling ambiguities due to unknown standard free energies of reaction barriers.

The accuracy increasing effect of RNA polymerase is unknown. It is here accounted for by a DNA context neutral enhancement of the specificity of cognate in relation to non-cognate base pair selections, in line with the accuracy enhancing features of ribosomal RNA during mRNA translation by tRNAs [11; 25](see also Discussion). For each type of nucleotide substitution error and DNA context, the accuracy of transcription, *A*, is determined by an initial selection parameter, *I*, and a proofreading selection parameter, *F*, as well as by the concentrations of the free nucleoside triphosphates ATP, UTP, GTP and CTP [26].

### Initial selection of nucleotides

Initial selection of incoming nucleotides takes place in the POST·NTP state, from which the initially associated nucleotide either dissociates from the TEC or is phosphodiester bonded to the nascent transcript with probability *P* ^*c*^ for a cognate and probability *P* ^*nc*^ for a non-cognate nucleotide (Fig. 1). The initial selection parameter *I* is defined as the ratio of the *k*_*cat*_ */K*_*m*_ values for initial transcript elongation with a cognate and a non-cognate nucleotide [13; 26; 27]. With equal association rate constants for cognate and non-cognate nucleotides, *I* is the ratio of *P* ^*c*^ and *P* ^*nc*^ [19; 27]:

$$I=\frac{{\left(k_{cat}/K_{m}\right)}^{c}}{{\left(k_{cat}/K_{m}\right)}^{nc}}=\frac{k_{a}^{c}}{k_{a}^{nc}}\frac{P^{c}}{P^{nc}}=\frac{P^{c}}{P^{nc}}$$ (1)

Expressing *P* ^*c*^ and *P* ^*nc*^ in terms of the elementary rate constants of transcript elongation gives:

$$I=\frac{P^{c}}{P^{nc}}=\frac{{\left(\frac{k_{c}}{k_{c}+q_{3}}\right)}^{c}}{{\left(\frac{k_{c}}{k_{c}+q_{3}}\right)}^{nc}}=\frac{1+{\left(\frac{q_{3}}{k_{c}}\right)}^{nc}}{1+{\left(\frac{q_{3}}{k_{c}}\right)}^{c}}$$ (2)

### Proofreading selection of nucleotides

The physical chemical principles of proofreading were initially described by Hopfield [28] and Ninio [29] and proofreading mechanisms were subsequently identified for aminoacylation of tRNA [30] and translation of mRNA by tRNA [31; 32]. After initial substrate selection, the accuracy of an enzymatic reaction can be amplified by a proofreading step in which non-cognate substrates are discarded from the enzyme with high probability in a thermodynamically driven dissociation step [19]. Transcriptional proofreading is here modeled as nascent RNA cleavage in a backtracked TEC, followed by dissociation of a 5’ dinucleotide containing the last incorporated nucleotide (Fig. 1A). The accuracy enhancement (*F*) by proofreading is the ratio of the probabilities for the correctly and the non-correctly incorporated nucleotides to escape proofreading dissociation and be secured by the next nucleotide incorporation. In terms of elementary rate constants of transcript elongation (Fig. 1), the proofreading parameter *F* is given by:

$$F=\frac{P_{F}^{c}}{P_{F}^{nc}}=\frac{1+{\left(\frac{\varsigma }{\kappa }\right)}_{F}^{nc}}{1+{\left(\frac{\varsigma }{\kappa }\right)}_{F}^{c}};{\left(\varsigma /\kappa \right)}_{F}^{c/nc}=\frac{k_{1}^{c/nc}}{k_{2}}\frac{1+\frac{q_{2}}{k_{3}\cdot [NTP_{n+1}]}\left(1+\frac{q_{3}^{c/nc}}{k_{c}^{c/nc}}\right)}{1+\frac{q_{1}{}^{c/nc}}{q_{c}}}$$ (3)

The four substrate selective rate constants in Eq. 3 are marked with *c*/*nc*. Cognate (*c*) and non-cognate (*nc*) rate constants may differ due to their different reaction activation barriers or differences between their initial and final reaction states. For example, during backward translocation a last incorporated cognate nucleotide moves from a template matched (PRE) to a template independent (BACK) state, while a non-cognate nucleotide moves from a template unmatched (PRE) to the BACK state (Fig. 1). Hence, we expect that *k*_*1*_^*c*^ < *k*_*1*_^*nc*^ and that *q*_*1*_^*c*^ < *q*_*1*_^*nc*^. Likewise, incorporation of a next cognate nucleotide is favored when the last incorporated nucleotide in POST·NTP is cognate rather than non-cognate and, hence, *q*_*3*_^*c*^ */ k*_*c*_^*c*^ < *q*_*3*_^*nc*^/ *k*_*c*_^*nc*^ (see Methods). The other four rate constants in Eq. 3 are neutral to the substrate identity. Note, however, that these neutral rate constants may greatly affect the value of the parameter *F* by determining the currently expressed fraction of its maximal value [13]. For instance, if *q*_*2*_/*k*_*3*_ is very small and *q*_*c*_ very large, the only accuracy contribution comes from *k*_*1*_^*c/nc*^ and if, in addition, *k*_*2*_ is very large then *F* ≈ 1 and there is virtually no accuracy amplification in the proofreading step.

The obligatory thermodynamic driving force, RT·log_e_γ, of the proofreading step [19] is here provided by the factor γ by which two free nucleoside triphosphates are shifted above equilibrium, *K*, with their corresponding free nucleoside monophosphates and pyrophosphate (*PP*_*i*_). The shift in equilibrium renders the reaction of phosphodiester bond formation practically irreversible (γ >> 1):

$$\frac{\left[NTP_{i}\right]\left[NTP_{i-1}\right]{\left[H_{2}O\right]}^{2}}{\left[NMP_{i}\right]\left[NMP_{i-1}\right]{\left[PP_{i}\right]}^{2}}=K\gamma$$ (4)

Although there is considerable experimental support for the existence of backtracking based transcriptional proofreading [17; 18; 23; 31; 32], quantitative studies of transcriptional proofreading are still lacking.

### Overall accuracy in selection of nucleotides

The overall accuracy *A* in the selection of the cognate nucleotide over a non-cognate nucleotide at any DNA template position is the ratio of the cognate and non-cognate substrate concentrations multiplied by the product of the initial and proofreading selection parameters. At equal cognate and non-cognate substrate concentrations, the normalized overall accuracy per substrate is given by:

$$A=I\cdot F=\frac{P_{I}^{c}P_{F}^{c}}{P_{I}^{nc}P_{F}^{nc}}$$ (5)

This relation (Eq. 5) holds for the competition between the cognate substrate and each one of its three competitors at each template position along the DNA genes of the chromosome. Taking intracellular substrate concentrations (*S* ^*c*^, *S* ^*nc1*^, *S* ^*nc2*^, and *S* ^*nc3*^) into account, the total accuracy *A*_*tot*_, defined as the per template site flow of product formation with a cognate substrate over the flow of product formation by all non-cognate substrates, is given by:

$$A_{tot}=\frac{[S^{c}]P_{I}^{c}P_{F}^{c}}{[S_{}^{nc1}]P_{I}^{nc1}P_{F}^{nc1}+[S_{}^{nc2}]P_{I}^{nc2}P_{F}^{nc2}+[S_{}^{nc3}]P_{I}^{nc3}P_{F}^{nc3}}$$ (6)

The probability of *any* misincorporation at a given template position is:

$$E={\left(A_{tot}+1\right)}^{-1}$$ (7)

By summation of the error probabilities of all template positions of a sequence, the expected number of errors per transcript is obtained, and division by the transcript length gives the error frequency.

No complete set of mismatched DNA/RNA nearest-neighbor parameters has been published. Therefore, we used the existing set of all A·A, C·C, G·G and T·U interactions [33] for all types of mismatches. For example, at a position with a T in the leading strand DNA, the correct RNA incorporation is a U, and the A in the complementary DNA strand can be mismatched with A, G or C. However, lacking the full dataset, the mismatch energies of the G and C mismatches are approximated by the A·A mismatch interaction energy but with the G and C nucleotide concentrations. Nucleotide concentrations used in calculations were 3560 μM ATP, 325 μM CTP, 1660 μM GTP and 667 μM UTP [34].

### Modeling the accuracy of ribosomal RNA transcription

**Fig 2:**
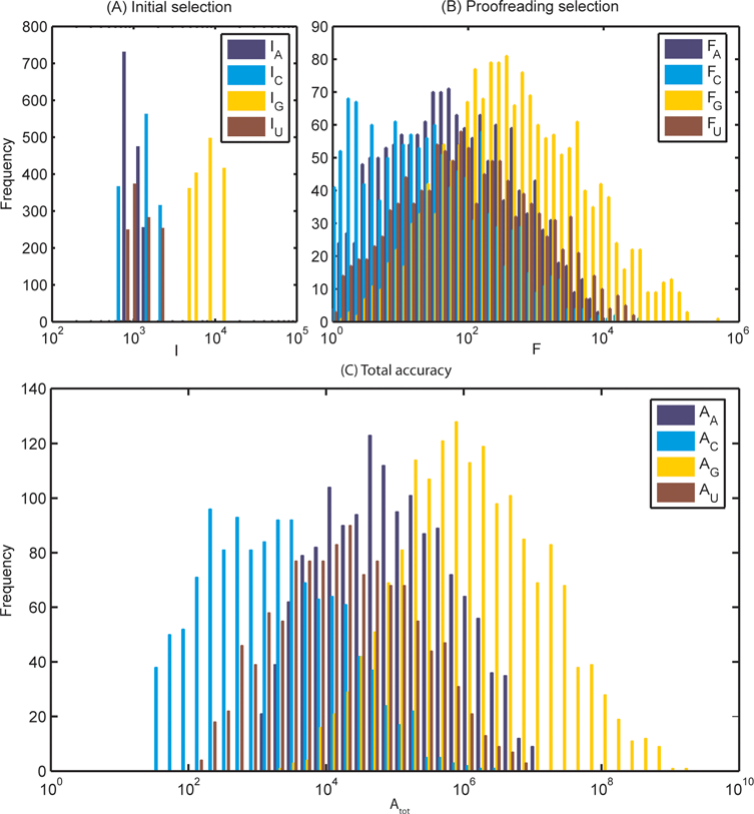
Histograms of initial selection, proofreading and total accuracy in *rrnC*, grouped after the cognate substrate. A) Initial selection. With only one type of mismatch per template (A·A, C·C, G·G or U·T), the scope of each bar represents the initial selection *I*_*X*_ of the correct nucleotide *X* against *Y* over the incorrect nucleotide *Y* against *Y*. The distribution is discrete, with 16 distinct levels of accuracy, although some mismatches are so similar in selection they appear in the same bar. B) Proofreading selection. The scope of each bar represents the proofreading *F*_*X*_ of the correct nucleotide *X* against *Y* over the incorrect nucleotide *Y* against *Y*. The distribution is near-continuous and slightly truncated at 1. C) Total accuracy, as defined in Eq. 6. The scope of each bar represents the total accuracy *A*_*X*_ of the correct nucleotide *X* against *Y* over the incorrect nucleotide *Y* against *Y*. The maximum of *A*_*tot*_ is a factor 5,500,000 larger than the minimum.

The ribosomal RNA operon C (*rrnC*) from *E*. *coli*, around 5,550 base pairs in length, was used to exemplify the DNA context effect on transcriptional accuracy. The results are presented as histograms showing the occurrence of positions with each level of accuracy (Fig. 2). The parameter choices are explained in the supporting information, and the conclusions from the results are shown to be robust with regard to parameter assumptions (see Methods).

To account for the accuracy enhancing effect of the polymerase itself, we assumed that phosphodiester bond formation in the initial selection and the incorporation following a misincorporation is 100 times slower for a non-cognate than for a cognate nucleotide, in line with previous suggestions [35] (see also Methods). The polymerase contribution to transcriptional accuracy is expected to vary with the type of mismatch, but the context dependent accuracy variation reported here, we deem to be a robust property of transcription (see Discussion).

### Modeling initial selection of nucleotides

The initial selection parameter *I* is discretely distributed (Fig. 2A), since its variation depends only on the identity of the complemented nucleotide and its nearest neighbor. Indeed, with fixed nucleotide concentrations the total initial selection per position, comparing the probability of the cognate incorporation to the probability of any of the three types of misincorporations, can take only 4^2^ = 16 values. With the present dataset on mismatch energies, initial selection varies by a factor 22 between its smallest and largest values.

### Modeling proofreading selection of nucleotides

In contrast to the initial selection parameter *I*, the proofreading selection parameter *F* has a near-continuous distribution. The reason is that it depends on a large number of nucleotides in the sequence of the transcription bubble. As seen in Eq. 3, *F* is determined by the reaction rate ratio (ς/κ)^c/nc^, that contains the eight discriminating rate constants *k*_*1*_^*c/nc*^, *k*_*c*_^*c/nc*^, *q*_*1*_^*c/nc*^ and *q*_*3*_^*c/nc*^, and their effect on *F* is tuned by the four non-discriminating rate constants *k*_*2*_, *k*_*3*_, *q*_*2*_ and *q*_*c*_. The context dependent variation of these twelve rate constants depends in turn on 11 of the 16 base pairs of the transcription bubbles involved in one round of proofreading (Fig. 2B), leading to 4^11^ different values of the proofreading selection. In our model, the five base pairs in the middle of the 16-nucleotide sequence do not affect the accuracy variation, since they and their nearest neighbors do not open or close during proofreading, only shift position. However, recent results from Bochkareva *et al*. [20] indicate that also these five base pairs affect the transcription rate, albeit for reasons that have remained unclear.

The proofreading part of the accuracy of the *rrnC* operon spans more than 5 orders of magnitude, with a factor of 430,000 between highest and the lowest values. Again, the model describes the sequence dependent part of proofreading with a small polymerase effect, meaning that the actual proofreading selection with catalytic and steric effects of the polymerase is likely to be higher, but the relative variation would be similar. In Fig. 2B, the span of the G·G proofreading is truncated at the minimum accuracy of 1, but with further amplified proofreading accuracy, the whole proofreading span would be even larger.

### Modeling total accuracy of nucleotide selection

The *A*_*tot*_ variation range, as a fraction of the maximum and the minimum, is for the *rrnC* operon 5,500,000, reflecting the context dependent contributions of initial selection, proofreading selection, and nucleotide concentrations (Eq. 6). The spectrum of *A*_*tot*_ resembles a log-normal distribution due to the near normally distributed standard free energies in the exponent of the Eyring equation. Convenient measures of the expected *A*_*tot*_ value, <*A*_*tot*_>, and its standard deviation *σ*_*Atot*_, are therefore the exponent of the average value of log_e_(*A*_*tot*_) and the exponent of the standard deviation of log_e_(*A*_*tot*_), respectively. By these measures we obtain for the *rrnC* operon <*A*_*tot*_> = 2.0·10^5^ and *σ*_*Atot*_ = 19.9. While the <*A*_*tot*_> value is sensitive to the magnitude of the RNA polymerase specific accuracy enhancement, *σ*_*Atot*_ and the total span of the *A*_*tot*_ variation are not (see Methods).

**Fig 3:**
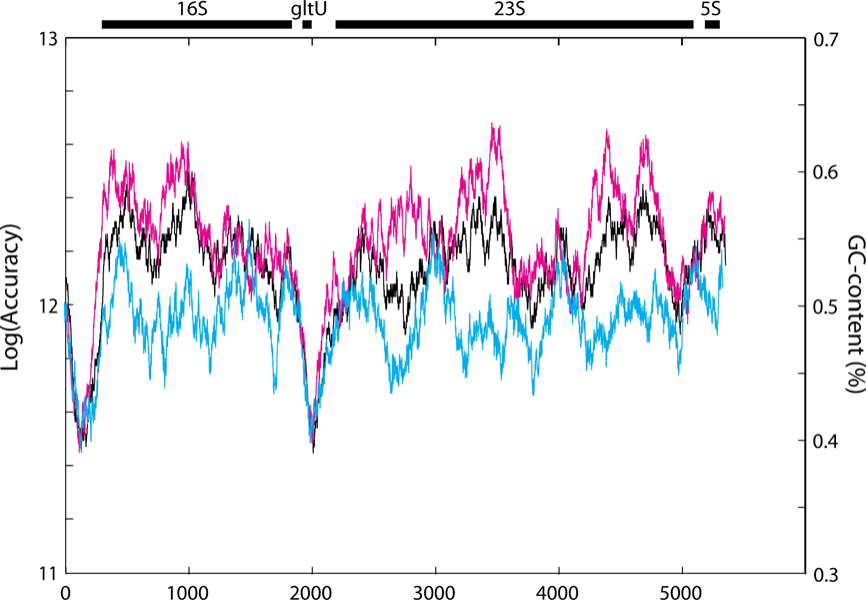
GC-content, total accuracy and transcriptional accuracy of antisense strand. Moving average over the *rrnC* operon with a window size of 200. The black curve represents the GC-content, to be compared to the right-hand side y-axis. The curves in magenta and cyan represent the logarithm of the accuracy of the *rrnC* transcript and its antisense strand, respectively. All curves drop at the linker regions before the 16S and 23S genes. The accuracy variation of both accuracy curves roughly follows the GC-content, but there is also additional variation. The accuracy of the antisense strand is lower by a factor 2.78, calculated as the exponent of the ratio of averages of the logarithm-transformed accuracy.

We compared a moving average of the GC-content of the *rrnC* operon to a moving average of the logarithm of the total accuracy, both with a window size of 200 (Fig. 3). It is seen that the total accuracy correlates positively with the GC-content, and also that some of the sequence dependent accuracy variation cannot be explained by GC-variation only. Indeed, both accuracy and GC-content are smaller in non-coding regions of the operon (upstream of the 16S and 23S genes). Furthermore, our model predicts the accuracy of transcription of the antisense strand of the *rrnC* operon, with the same GC-content as the sense strand, to have an about three-fold lower <*A*_*tot*_> value than the sense strand. From these findings we suggest that the transcriptional accuracy is significantly higher for template sequences encoding functional RNA than for those encoding non-functional RNA. By inference this could also mean that transcription of open reading frames is more accurate than transcription of non-coding RNA.

To identify the high and low accuracy motifs predicted by our model, a dataset of all the 4^11^ possible transcription bubble variants was constructed. The theoretical range of transcriptional accuracy from this set is about 73,000,000, calculated as the ratio of the maximum value of 2.5·10^9^ and the minimum value of 35. The sequences representing these extreme values are the hyper-accurate TATAGGNNNNNAGTCA and the error-prone GCGTTTNNNNNGCATT sequences. The top ten accuracy and error-prone motifs are presented in Table 1.

**Table 1:** The top ten of error-prone and high-accuracy motifs.

Low accuracy motifs		High accuracy motifs	
Motif	Accuracy	Motif	Accuracy
GCGTTTNNNNNGCATT	34.6669	TATAGGNNNNNAGTCA	2.5473·10^9^
CGCTTTNNNNNGCATT	34.6703	ATAAGGNNNNNAGTCA	2.4079·10^9^
GCGTTTNNNNNGCATG	34.6853	ATATGGNNNNNAGTCA	2.3814·10^9^
CCGTTTNNNNNGCATT	34.6854	AATAGGNNNNNAGTCA	2.3703·10^9^
GGCTTTNNNNNGCATT	34.6862	TTAAGGNNNNNAGTCA	2.3668·10^9^
CGCTTTNNNNNGCATG	34.6901	TTATGGNNNNNAGTCA	2.3114·10^9^
GCGTTTNNNNNGCAAG	34.6921	TATAGGNNNNNAGTCC	2.2379·10^9^
CGCTTTNNNNNGCAAG	34.6974	CTAAGGNNNNNAGTCA	2.2343·10^9^
GCGTTTNNNNNGCAAT	34.6993	ATTAGGNNNNNAGTCA	2.2231·10^9^
GCGCTTNNNNNGCATT	34.7017	GATAGGNNNNNAGTCA	2.1790·10^9^

**Fig 4:**
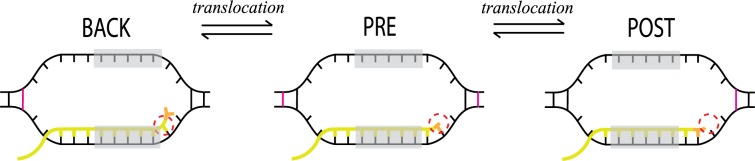
The transcription bubble in three states. The active site position is circled, RNA is in yellow with the last incorporated nucleotide in orange, and the borders of the affected DNA base pairs are in magenta. With a transcription bubble size of 12 bp, 16 bp of DNA are melted or adjacent to a melted base pair during one elongation step. However, 5 bp (shaded) only shift position during these translocations, and are never part of any changes in binding status, resulting in 11 bp affecting the accuracy.

The ten most error-prone motifs and the ten motifs with the highest accuracy of all possible 11-base pair transcription bubbles (5’ to 3’ DNA). Five bases in the middle of the transcript are arbitrary, as explained in Fig. 4. The underlined nucleotide is the position subject to fidelity control.

From the sequences of these high and low accuracy motifs we propose, firstly, that when there are weak interactions between the DNA strands at the upstream end of the transcription bubble (to the left in the bubbles in Fig. 4) and strong interactions at the downstream end, backward translocation is facilitated and forward translocation obstructed. This leads to high accuracy amplification by proofreading (*F*) and high total transcriptional accuracy (*A*). Secondly, when the RNA/DNA interactions that re-form at the exit end of the hybrid upon backtracking (the left-hand end in Fig. 4) are strong, and the RNA/DNA interactions at the active site end are weak, backtracking is facilitated and forward translocation obstructed, again supporting accuracy amplification by proofreading. In conclusion, high stability in the 5’ end and low stability in the 3’ end of the coding template DNA, and low stability in the 5’ end and high stability in the 3’ end of the transcript give a high accuracy, and vice versa.

### Experimental validation of the accuracy model

Transcriptome errors were recently assessed for the round worm *C*. *elegans* by Gout *et al*. [4]. The transcription errors were estimated by a single molecule approach effectively removing interference from reverse transcription and PCR errors. The fragmented transcriptome was reverse transcribed three times, and each cDNA sequenced. From 0.5% (180,000) of the original number of fragments, they obtained two or more cDNAs of sufficient quality per individual RNA fragment. In these, they found 83 authentic transcription errors as validated by their presence in more than one cDNA from the same individual transcript fragment.

**Fig 5:**
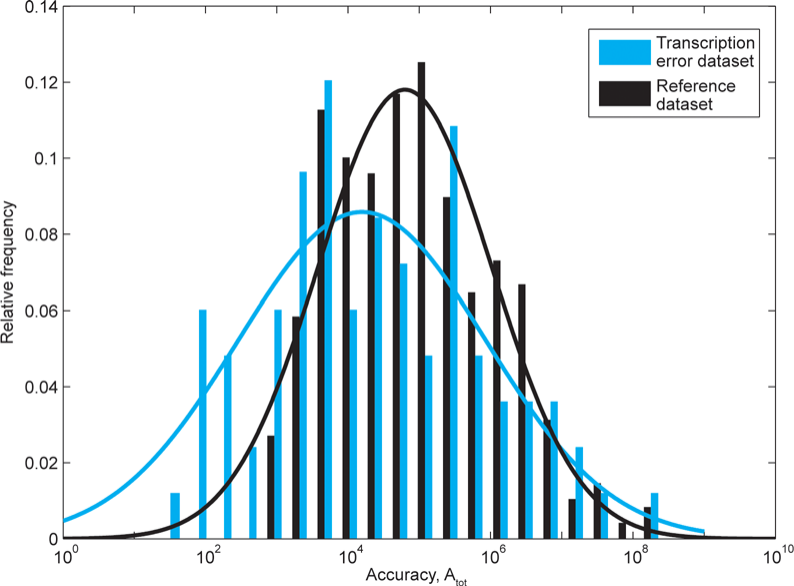
Predicted accuracy in the transcription error (blue) and reference (black) datasets. The transcription error dataset consists of the transcription errors found by Gout *et al*. [4], and the 479 reference positions were chosen randomly from transcripts in the *C*. *elegans* reference genome database (Ensemble release 66). The accuracy, calculated using the same parameters as in *E*. *coli*, is presented as a histogram of relative frequencies in the two datasets. The blue and black lines are normal distributions fitted to the histograms of the datasets, emphasizing that the errors furthest to the right represent extreme error positions, and that many of the error sequences are more error prone than an average motifs. The two distributions are significantly different (p = 2.03·10^-5^).

The sequence of the transcription bubble around each of these error positions was extracted from the *C*. *elegans* reference genome (Ensembl release 66, as used in [4]), together with transcription bubble sequences around 479 random positions in 20 arbitrarily chosen reference transcripts (intron positions excluded) (S1 Table). We then used our transcription model to compare its predicted errors for the “error dataset” (blue staples in Fig. 5) and the “reference dataset” (black staples in Fig. 5). It is seen that the accuracy spectrum from the error dataset is shifted to the left and thus has lower accuracy and higher errors compared to the normal distribution fitted to the reference data. Indeed, the <*A*_*tot*_> value for the error dataset is about four times smaller than that for the reference dataset. A one-sided Student’s t-test applied to the approximately normal *log(A*_*tot*_) spectra in Fig. 5 shows that the probability that they have the same probability distribution (p-value) is 2.0·10^-5^. From this encouraging result we suggest that our accuracy model correctly reproduces major aspects of the accuracy tuning effects of the DNA template motifs in the ever moving accuracy bubble (see Discussion). A more detailed comparison of error sets with reference datasets will require extended knowledge of putative biases in the error detection procedures used by Gout *et al*. [4].

## Discussion

We have modeled the expected variation in transcriptional accuracy due to the context dependent variation of the standard free energy of the transcription bubble during the movement of the RNA polymerase. The modeling approach is illustrated by the prediction of the accuracy variation during transcription of the ribosomal RNA operon C (*rrnC*) in *E*. *coli*. The origin of the free energy variation is the changing composition of pairs of DNA/DNA or DNA/RNA base pairs in the transcription bubble. Initial selection of nucleotides before phosphodiester bond formation displays a discrete accuracy spectrum (Fig. 2A), while the proofreading accuracy spectrum is near continuous (Fig. 2B), making the overall accuracy spectrum remarkably broad (Fig. 2C). The total accuracy, both in *rrnC* and in the dataset containing all 4^11^ possible accuracy determining motifs, resembles a log-normal distribution due to a near normal distribution of the rate determining standard free energies in the transcription bubble and nascent transcript. Thus the logarithm of the maximal and minimal accuracies have approximately the same distance from the expected value, while the accuracy distribution is very skewed to the right on a linear scale (Fig. 2C). The proofreading accuracy of a sequence motif is inversely correlated to transcription rate, since high accuracy requires a slowly transcribed sequence that is prone to pausing and backtracking.

These results suggest that, due to DNA context, transcription errors may vary between very high and very low levels. Accordingly, our data challenge the common views that transcription errors always are in a low range between 10^-5^ and 10^-4^ [1] or lower [4], and that mRNA translation errors always dominate over transcription errors regarding amino acid substitution mistakes in intracellular proteins. Instead, transcription errors are expected to dominate over translation errors in some contexts, where transcriptional errors would have a much higher biological impact than previously imagined. In addition, the right-skewed accuracy distribution suggests frequently occurring high error sites in transcripts. The existence of transcriptional error rates that supersede the translational error rates is thus a robust conclusion from the experimental average error frequency of transcription together with the error variation predicted by the present modeling. Yet, the precise extent of dominating transcriptional errors will depend on the largely unknown variation of translational errors [3] and the so far unknown RNA polymerase contribution to transcriptional accuracy. The strong template dependent accuracy variation we have identified could be yet another driving force for the choice of nucleotides in functional RNA molecules and significantly affect synonymous codon usage in mRNAs [36].

### Robustness of results

The validity of our model for transcriptional error variation rests upon a set of simplifying assumptions. We have derived the context dependent variation in transcription errors from nearest-neighbor parameters measured in solution, in the absence of RNA polymerase. Assumptions here are that the base stacking between pairs of correct and incorrect base pairs in solution are similar to those in the catalytic site of the RNA polymerase (see Methods).

Although the RNA polymerase is likely to significantly amplify the accuracy of transcription in a mismatch dependent manner, we suggest that it is the energy variation from the DNA context around the active site that constitutes the largest part of the sequence dependent accuracy variation. Presently, our model depends on an incomplete mismatch nearest-neighbor parameter dataset. The missing part of the complete set has been measured, but the results have remained unavailable [33]. Accordingly, each template base identity is now tested against only one type of non-cognate nucleotide, instead of three. It may be that use of a complete dataset would not substantially alter the range of the accuracy variation, since the interaction energies of the incorrect base pairs would still be compared to the same cognate base pair energies and the effect of the non-discriminating reactions on the proofreading selection would remain unaltered. After all, the mismatch is only one out of the 11 base pairs affecting the accuracy variation. All the same, if the missing data set is made available it is likely to improve significantly our model predictions.

The reactions are treated as if the stability of transition states intermediates are predictable and without sequence-specific effects, which allows us to choose constant transition state barriers. If this were not true, it would deflate our predictions about sequence motifs, but the main result based on the variation in sequence energy between different transcription bubbles would persist as long as the polymerase has not evolved to attenuate this variation.

### Experimental outlook

The most direct and reliable validation of the present predictions of large context dependent variation of transcriptional errors requires transcriptional error measurements in different template contexts of similar quality as the newly developed system for studies of mRNA translation errors on the ribosome [3]. Recent developments in this field by Imashimizu *et al*. [2], applied to transcription errors in E. coli, bring great promise for future investigations. However, these detections of transcription errors might still be partially obscured by sequencing and/or reverse transcription errors, and we have therefore chosen not to use their dataset for model validation [2].

On the other hand, while the method developed by Gout *et al*. [4] was not used to investigate the error frequency for each position of the *C*. *elegans* transcriptome, it was reliable for detection of true transcriptional errors and therefore proved useful to us for comparison and validation of our model. The model could accurately predict an ensemble of higher error probability motifs in the experimental dataset of transcription errors, but also predicted a disproportionate fraction of transcription errors in high-accuracy positions. Apart from these two studies [2; 4], there are no experimental measurements of the context dependent accuracy variation under *in vivo* conditions to use for model validation, but the recent development brings hope for the future.

The model would also benefit from more information about nucleotide-specific variations of interactions with the polymerase. The contribution of the polymerase itself to the selectivity of base incorporation was here assumed to be neutral, providing the same discrimination factor of 100 to all types of mismatches. It will be of great interest to investigate how the transcriptional machinery copes with high error as well as high accuracy hotspots, since accuracy enhancement in both initial selection and proofreading is at the cost of greatly reduced transcription efficiency by the rate-accuracy trade-off [3; 13]. An interesting question is therefore if RNA polymerases have evolved to discriminate strongly against the errors that are most likely to occur, or against the high-accuracy positions that may induce pauses. Another testable prediction of our model is that the proofreading parameter *F* will decrease with increasing NTP concentration, as seen in Eq. 3, since elevated NTP levels drive the POST complex in Fig. 1A towards the POST·NTP complex and phosphodiester bond formation, thereby preventing translocation to the PRE-state, backtracking and nucleotide release.

### Evolutionary adaptations

The logic behind the accuracy variation in sequence motifs suggests that adaptations to increase the transcriptional accuracy are complex. That is, an error-prone motif might be part of a hyper-accurate motif a few translocations later, since the same base pairs that made an earlier transcription bubble accurate will make a later bubble inaccurate when the base pairs have shifted to its other end. This makes it difficult to design globally super-accurate DNA sequences and also to predict evolutionary adaptation in sequence data. It is however likely that some motifs are counter-selected locally, at positions where accuracy or speed is critical. The predicted higher accuracy in the coding sequence of the *rrnC* operon (Fig. 3), compared both to linker regions and the antisense strand, suggests an adaption of the coding strand sequence to increase the accuracy of transcription and the quality of the transcription product, but can also be a side-effect of the selection of bases in the coding sequence for other functions. In conclusion, our results predict very large and DNA context dependent variation of transcriptional errors. This suggests that transcriptional errors may dominate over other sources of amino acid substitution errors in the proteome in some, but not other contexts. It also raises questions about how the quality of large functional RNA molecules like ribosomal RNA is maintained by the transcription machinery.

## Methods

All calculations were performed in MATLAB 7.9.0 (The MathWorks, MA, USA). The *rrnC* DNA sequence was downloaded from EcoCyc [37], between positions 3,939,539 and 3,945,090. The operon was chosen because the transcription of rRNA is well studied and not affected by trailing ribosomes.

### The transcription bubble

The model describes profound effects of the template context dependent free energies of pairs of base pairs in the transcription bubble on the kinetics of transcript elongation. The nearest-neighbor parameters for double stranded DNA [9] and RNA/DNA hybrid [10] are estimated from the melting energies of the sequences in solution, and include the stacking effect for all combinations of adjacent base pairs.

Each state in the transcription model is represented by an elongation complex that consists of an RNA polymerase, a transcription bubble in the double-stranded DNA and a nascent RNA transcript. The total free energy of the elongation complex, the state energy, is the sum of the energy costs of breaking up the DNA double helix to form the transcription bubble, $\Delta G_{DNA/DNA}^{0}$, the remediating formation of RNA/DNA hybrid, $\Delta G_{RNA/DNA}^{0}$, and of the interactions between the polymerase and the nucleotide sequences, $\Delta G_{pol}^{0}$[7].

$$\Delta G_{state}^{0}=\Delta G_{DNA/DNA}^{0}+\Delta G_{RNA/DNA}^{0}+\Delta G_{pol}^{0}$$ (8)

The nearest-neighbor model predicts the melting energy of a sequence by summation of the nearest-neighbor parameters for the constituent base pairs, with one parameter for every pair of two adjacent base pairs [9]. The nearest-neighbor parameter represents the melting energy of the two base pairs including their mutual base stacking effect. Each non-terminal base pair in the sequence contributes to its total melting energy through two nearest-neighbor parameters, being part of two pairs of base pairs.

The free energy of transcription bubble formation at each position in the transcribed gene is hence the sum of the nearest-neighbor parameters of the 13 pairs of base pairs formed from 12 denaturated base pairs [9]. In the ends of the bubble, we use half of the value of the parameter for the half-denaturated nucleotide pair with the last opened and first intact base pairs (Fig. 4).

The formation energy of the base pairing in the RNA/DNA transcript is predicted in a similar fashion, using nearest-neighbor parameters for RNA/DNA base pairs [10]. The length of the hybrid is 8 or 9 base pairs, giving 7 or 8 pairs of base pairs with corresponding nearest-neighbor parameters (Fig. 4). For pairs of hybrid base pairs with a nucleotide mismatch, mismatch nearest-neighbor parameters are used instead [33].

The accuracy calculations, however, are based on the difference in free energies between adjacent states of the elongation complex. Since adjacent states have partly overlapping transcript bubbles, parts of the nucleotide sequence energies will cancel out in the comparison, so that not all of the nearest-neighbor parameters in the transcription bubble appear in the resulting accuracy estimates.

The extension of the melting energy data to the nucleic acid sequences of the transcription bubble inside the RNA polymerase involves some assumptions. First, to predict the energies of hydrogen bonds between bases using free solution parameters, we make the assumption that these energies are an inherent property of the double-stranded sequences that is preserved even within the polymerase. Since this is the energy of the hydrogen bond upon entry to the polymerase, the polymerase *must* at some point apply this energy to break the bond, even though it is possible that weakening of bonds through an applied force occurs at a different reaction step than the final break.

Second, we also assume conservation of base stacking effects in the reactions of transcript elongation. These stacking effects are unique to double-stranded sequences, which to a large extent keep their structure.

Third, the effects of the polymerase are part of all reaction rates through the transition state barriers of the reactions, through catalysis of phosphodiester bond formation and cleavage, and naturally by DNA sliding in the translocation reactions. However, any sequence specific effects of the polymerase are neglected, so that in comparison between two different state energies the polymerase effects cancel out, and only the transition state barriers and base pair composition differences remain (Eq. 8). The transition state barriers in the model are assumed to be uniform for all substrates. The rationale is that the polymerase interacts only with the backbone of the sequences, since the nucleobases face each other, and effects that are not sequence-specific will cancel out in comparison between transcription bubbles.

This is likely an over-simplification. Experimental evidence suggests, for instance, that the polymerase might interact with the hybrid sequence [20], and also that different nucleotides are added at different rates, irrespective of nucleotide concentration [38; 39], only to mention a couple of examples of possible polymerase-sequence interactions. The second example indicates that the stereochemistry in the transition states could be different for different base pairs. However, as we lack sufficient and reliable information of this kind of effects to include in the model, we instead make the simplifying assumption that the effects are negligible to the accuracy and cancel out in comparison between cognate and non-cognate substrates. In any case, the sequence-dependent accuracy variation that we do predict would persist even with additional sources of variation, if they do not specifically counteract sequence stability effects. One neglected factor that could have such additional sequence-specific effects is secondary structures of the transcript.

### Reaction rate constants

The reaction rate constants within elongation steps are denoted *k*_*i*_ and *q*_*i*_ (Fig. 1A). According to the Eyring equation, each constant *k* or *q* can generically be written as the product of a pre-factor constant *k*_*pre*_ and the exponential of the difference in standard free energy (ΔG) between the transition state, marked ‡, and the initial state:

$$\text{reaction rate constant}=k_{pre}e^{-\left(\Delta G^{‡}-\Delta G^{0}\right)/RT}$$ (9)

Here, R is the gas constant (8.314510 J·K^-1^·mol^-1^), T the absolute temperature (310 K) and *k*_*pre*_ is set to 10^9^ s^-1^, in line with earlier studies [40]. In addition to depending on the transition state barriers, the rate of a reaction from an initial state to a final state also depends on the difference in free energy between them. The logic is that the rate constant from the state with highest free energy is always given only by the reaction barrier, and the rate constant from the state with lowest free energy by the barrier plus the free energy difference (ΔΔG) between final and initial states, as illustrated in S1 Fig. In the nucleotide addition cycle (Fig. 1A), five cognate rate constants, *k*_*1*_, *k*_*2*_, *q*_*1*_, *q*_*2*_ and *q*_*3*_, depend on the standard free energy difference between initial and final reaction states, as well as *k*_*c*_ in the non-cognate case. The accuracy variation predicted by our model emerges from the template context dependent variation in ΔΔG for cognate and non-cognate substrates, while transition state barriers and *k*_*pre*_ are assumed to be template context independent unless otherwise stated.

As mentioned above, only some of the reactions discriminate against errors: rate constants *k*_*c*_ and q_3_ confer the intrinsic selectivity of initial selection (Eq. 2) and rate constants *k*_*c*_, *q*_*3*,_ *q*_*1*_ and *k*_*1*_ confer the intrinsic selectivity of proofreading selection (Eq. 3). The effect of the non-cognate substrate compared to the cognate substrate is a reduction in bubble stability, or an increase in the free energy of the TEC (S2 Fig.). Most misincorporations will nevertheless stabilize the complex, compared to an empty active site as in state POST, but in a few cases the misincorporation weakly destabilizes the complex [33]. The destabilizing energy difference is included in the phosphodiester bond formation energy barrier, with the consequence that phosphodiester bond formation discriminates with a sequence dependent component in these cases. This is in addition to the polymerase effect, implemented as a larger energy barrier for phosphodiester formation for non-cognate than for cognate substrates, which confers a context independent factor of 100 in advantage to the cognate reaction. All rate constants except *k*_*c*_, *q*_*3*,_ *q*_*1*_ and *k*_*1*_ are the same for cognate and non-cognate nucleotides, and rate constants *q*_*c*_ and *k*_*3*_ do not vary with template context.

All model input parameters, including the transition state barriers, are summarized in Table 2. The transition state barriers are tuned so that the total transit time through the *rrnC* operon matches the experimental measure of approximately 1 minute [41] and that proofreading contributes significantly to the accuracy of nucleotide selection, in accordance with experimental observations [23].

**Table 2:** Parameters in the calculations.

Parameter	Description	Value	Measured or tuned	Reference
[ATP], [CTP], [GTP], [UTP]	*In vivo* concentrations	See reference	Measured	[34]
10 DNA/DNA	Nearest-neighbor parameters	See reference	Measured	[10]
16 RNA/DNA	Nearest-neighbor parameters	See reference	Measured	[10]
32 Mismatch	Nearest-neighbor parameters	See reference	Measured	[33]
k_pre_	Pre-factor	10^9^ s^-1^	From reference	[40]
k_pre-a_	Pre-factor of association	6.4·10^11^ M^-1^s^-1^	Tuned	
ΔG_a_	Reaction energy barrier of association and dissociation	10·RT Jmol^-1^	Tuned	
ΔG_c_	Reaction energy barrier of phosphodiester bond formation	13·RT Jmol^-1^	Measured + tuned	[42]
ΔG_translocation_	Reaction energy barrier of translocation	11·RT Jmol^-1^	Tuned	
ΔG_cut_	Reaction energy barrier of transcript cleavage	18·RT Jmol^-1^	Measured + tuned	[43]
ΔG_forward bias_	Added stability to post-translocated states	2.5·RT Jmol^-1^	Tuned	
Polymerase effect	Mismatch discrimination by the polymerase	100	Tuned	

Summary of all input information used in the calculations. R is the gas constant, 8.314510 J·K^-1^·mol^-1^, and T is the temperature 310 K.

In detail, all reaction rate constants in the nucleotide addition cycle (Fig. 1A) were calculated as follows:

*k*_*3*_ is the second-order reaction rate constant for binding of nucleotide *i* to the polymerase, and is thus multiplied by the nucleotide concentration, [NTP_i_], of the incoming nucleotide. The pre-factor of association, *k*_*pre-a*_, is given by *k*_*pre*_/<[NTP]> = 6.4·10^11^ M^-1^s^-1^, where <[NTP]> is the averaged nucleotide concentration. The rate constant *k*_*3*_ is formulated in accordance with the Eyring formalism as a second order pre-factor multiplied by an exponential, *k*_*pre-a*_·exp(-ΔG_a_/RT). The free energy reaction barrier of nucleotide association, *ΔG*_*a*_, is set to 10·RT J·mol^-1^, so that *k*_*3*_ becomes 2.9·10^7^ M^-1^s^-1^. Note that in proofreading selection (Eq. 3), *k*_*3*_ is multiplied by the concentration of the *next incorporated* cognate nucleotide.

*q*_*3*_ is the rate constant for nucleotide dissociation from the polymerase: *q*_*3*_ = *k*_*pre*_·exp(-(ΔΔG_NTP_ + ΔG_a_)/RT) s^-1^. The free energy reaction barrier used is *ΔG*_*a*_, the same as for *k*_*a*_, but there is also the difference in free energy between the POST state with free nucleotide and the state POST·NTP, ΔΔG_NTP_. When positive, ΔΔG_NTP_ is the stabilizing energy of the extra bound nucleotide by one additional nearest-neighbor parameter. When ΔΔG_NTP_ is negative, it is set to zero and the destabilizing effect of the incoming nucleotide instead becomes part of the catalytic rate constant *k*_*c*_ for phosphodiester bond formation (Fig. 1, see also next paragraph). Whether the intrinsic selectivity of the enzyme with respect to cognate and non-cognate nucleotides resides in the dissociation or forward rate constant makes no difference for accuracy calculations, since they depend on the ratio *q*_*3*_/*k*_*c*_ (Eq. 2).

*k*_*c*_ is the rate constant for phosphodiester bond formation. For cognate reactions, *k*_*c*_ = k_pre_·exp(-ΔG_c_/RT) = 2.3·10^3^ s^-1^, with ΔG_c_ = 13·RT. We assume that the cognate nucleotide is perfectly positioned for phosphodiester bond formation in the state POST·NTP, with base-to-base interactions already formed, which is why the rate is slightly higher than the *in vitro* experimental rate of 1200 s^-1^ [42]. For non-cognate reactions, *k*_*c*_ = (1/100)·*k*_*pre*_·exp(-ΔG_c_/RT) = 2.3·10^1^ s^-1^ when ΔΔG_NTP_ > 0 and *k*_*c*_ = *k*_*pre*_·(1/100)·exp(-(-ΔΔG_NTP_ + ΔG_c_)/RT) when ΔΔG_NTP_ < 0.

*k*_*1*_ is the rate constant for backward translocation from the state PRE to the state BACK and is given by *k*_*pre*_·exp(-(ΔΔG_BACK-PRE_ + ΔG_translocation_)/RT) s^-1^ when ΔΔG_BACK-PRE_ > 0 (PRE is more stable than BACK) and by *k*_*pre*_·exp(-ΔG_translocation_/RT) s^-1^ when ΔΔG_BACK-PRE_ < 0 (BACK is more stable than PRE). This means that *k*_*1*_ will differ for cognate and non-cognate elongation complexes only when BACK has a higher free energy than the cognate state PRE. The translocation reaction barrier ΔG_translocation_ is set to 11·RT J·mol^-1^, in line with experimental data [14; 24]. Like all translocation events, *k*_*1*_ comprises several changes in the double-stranded nucleic acids in the elongation complex: the double-stranded DNA opens one base pair in the direction that the polymerase travels and anneals one base pair in the other end, while the last incorporated base in the RNA transcript is shifted out of the active site into the backtrack binding pocket [21], breaking the hydrogen bonds to the opposite template base, and at the same time, one base pair at the other end of the RNA/DNA hybrid is re-annealed upon re-entry into the hybrid channel. These four sequence changes, opening or annealing one base pair in each end of the DNA/DNA and the RNA/DNA double helixes, make *k*_*1*_ depend on the base identity of no less than nine base pairs (the affected base pairs and their nearest neighbors).

*q*_*1*_ is the rate constant of the forward translocation from BACK to PRE, the reverse of *k*_*1*_, and is given by *k*_*pre*_·exp(-(ΔΔG_PRE-BACK_ + ΔG_translocation_)/RT) s^-1^ when ΔΔG_PRE-BACK_ > 0 and by *k*_*pre*_·exp(-ΔG_translocation_/RT) s^-1^ when ΔΔG_PRE-BACK_ < 0. *q*_*1*_ will hence differ for cognate and non-cognate cases when BACK has a lower free energy than the non-cognate state PRE, which is typically the case since the non-cognate PRE state RNA/DNA hybrid contains one misincorporation while the BACK hybrid is correct.

*q*_*c*_ is the rate constant of the factor-assisted hydrolysis of the phosphodiester bond just upstream of the active elongation site and is given by *k*_*pre*_·exp(-ΔG_cut_/RT) = 15 s^-1^, where ΔG_cut_ is set to 18·RT J·mol^-1^. Experimental data on the GreA-assisted hydrolysis reaction (*q*_*c*_) have been obtained under conditions different from those of the other parameters, but indicate that the chosen reaction rate is reasonable [43]. There is limited information suggesting a sequence dependent variation of *q*_*c*_ [23; 44], which has here been neglected.

*k*_*2*_ is the rate constant of forward translocation from PRE to POST, and is similar to the other translocation rate constants described above. It is given by *k*_*pre*_·exp(-(ΔΔG_POST-PRE_ + ΔG_translocation_)/RT) s^-1^ when ΔΔG_POST-PRE_ > 0 and *k*_*pre*_·exp(-ΔG_translocation_/RT) s^-1^ when ΔΔG_POST-PRE_ < 0. The difference from the reaction between states PRE and BACK is that the RNA/DNA hybrid in POST is only 8 base pairs long, preparing the active site for nucleotide association, so there are three instead of four sequence changes. None of these changes concern the last incorporated nucleotide, and *k*_*2*_ is hence indifferent to mismatches.

Another consequence of the shorter hybrid sequence is that the POST state is generally considerably less stable than the state PRE due to the longer hybrid sequence. Still, the net movement of the polymerase must be in the forward direction and the translocation fast enough to match the experimental transit time over the operon [41]. To solve this, we have used a forward bias, ΔG_forward bias_, that stabilizes the two forward-translocated states by 2.5·RT J·mol^-1^, meaning that, in equilibrium, the polymerase favors POST over PRE. This stabilization together with the irreversibility of the phosphodiester bond formation (Eq. 4) ensures the net forward motion of sufficient speed. ΔG_forward bias_ is included in the free energy differences between states PRE and POST, ΔΔG_POST-PRE_ and ΔΔG_PRE-POST_.

*q*_*2*_ is the rate constant of backward translocation from POST to PRE, the reverse of *k*_*2*_, and is given by *k*_*pre*_·exp(-(ΔΔG_PRE-POST_ + ΔG_translocation_)/RT) s^-1^ when ΔΔG_PRE-POST_ >0 and *k*_*pre*_·exp(-ΔG_translocation_/RT) s^-1^ when ΔΔG_PRE-POST_ < 0.

### Mean-time calculations

The above reaction rates describe the rates between the “internal” substeps of protein elongation. All internal reaction steps have the same total transcript length, and together constitute one elongation step. The rates *κ* and *ς between* elongation steps, i.e., the rates at which the transcript grows or is shortened, demand another method.

The reaction rate constants between elongation steps are obtained from the numeric integration of the master equation describing our model. One elongation event, passing through the internal states presented in Fig. 1, is described mathematically by the time derivative of the probability of being in each of the elongation states. When integrated, they express the mean time τ the polymerase will spend in each state (Eq. 10). The boundary conditions are given by our defining of the PRE state as the state which marks the limits of the distinctive elongation steps (Fig. 1), the start of transcription at the promoter site (from which the polymerase cannot backtrack) and the end at the termination site:

$$\begin{array}{l} \frac{dP_{\text{PRE}}}{dt}=-(k_{2}+k_{1})\cdot P_{\text{PRE}}+q_{1}\cdot P_{\text{BACK}}+q_{2}\cdot P_{\text{POST}}\Rightarrow \\ -1=-(k_{2}+k_{1})\cdot \tau_{\text{PRE}}+q_{1}\cdot \tau_{\text{BACK}}+q_{2}\cdot \tau_{\text{POST}}\\ \frac{dP_{\text{POST}}}{dt}=-(q_{2}+k_{3})\cdot P_{\text{POST}}+k_{2}\cdot P_{\text{PRE}}+q_{3}\cdot P_{\text{POST}\cdot \text{NTP}}\Rightarrow \\ 0=-(q_{2}+k_{3})\cdot \tau_{\text{POST}}+k_{2}\cdot \tau_{\text{PRE}}+q_{3}\cdot \tau_{\text{POST}\cdot \text{NTP}}\\ \frac{dP_{\text{BACK}}}{dt}=-(q_{1}+q_{c})\cdot P_{\text{BACK}}+k_{1}\cdot P_{\text{PRE}}\Rightarrow \\ 0=-(q_{1}+q_{c})\cdot \tau_{\text{BACK}}+k_{1}\cdot \tau_{\text{PRE}}\\ \frac{dP_{\text{POST}\cdot \text{NTP}}}{dt}=-(q_{3}+k_{c})\cdot P_{\text{POST}\cdot \text{NTP}}+k_{3}\cdot P_{\text{POST}}\Rightarrow \\ 0=-(q_{3}+k_{c})\cdot \tau_{\text{POST}\cdot \text{NTP}}+k_{3}\cdot \tau_{\text{POST}} \end{array}$$ (10)

The elongation rates *κ* and *ς*, at which the TEC moves forward and backward in a series of elongation events, are provided by the rates *k*_*c*_ and *q*_*c*_ at which the forward and backward reactions occur given that the TEC inhabit the appropriate state. This probability of inhabitation is expressed as the fraction of the mean time spent in the associated state (POST∙NTP for *κ* and BACK for *ς*) and the total mean time of this elongation step; that is, the sum of the mean times of all the states:

$$\kappa =k_{c}\frac{\tau_{\text{POST}\cdot \text{NTP}}}{\tau_{\text{BACK}}+\tau_{\text{PRE}}+\tau_{\text{POST}}+\tau_{\text{POST}\cdot \text{NTP}}},\varsigma =q_{c}\frac{\tau_{\text{BACK}}}{\tau_{\text{BACK}}+\tau_{\text{PRE}}+\tau_{\text{POST}}+\tau_{\text{POST}\cdot \text{NTP}}}$$ (11)

Solving Eq. 10 and 11 bestows us with the mean times in terms of the reaction rates, which are dictated by the differences in free energy between states and the related energy barriers as described above.

### Accuracy calculations

Using the above methods, the reaction rates can be calculated, and from that the normalized accuracy according to Eq. 2, 3 and 5. The key result of the large accuracy variation is quite robust to changes in parameters, even at other transcription rates, as demonstrated in S3 Fig.

The primary action of the so far outlined proofreading mechanism begins with a correctly or incorrectly incorporated base in the pre-translocated state. Testing the base by proofreading occurs at least once per transcript elongation cycle. However, following nucleotide cleavage, the nucleotide that is now the last nucleotide in the post-cleavage transcript undergoes another round of proofreading that starts in the post-translocated, instead of in the pre-translocated, state (Fig. 1). This and other secondary proofreading effects of transcript cleavage generally confer but a small accuracy enhancing effect. They strongly depend on parameter choice, nucleotide concentration and an extension of the proofreading motif to the next two transcription bubbles, and are therefore not discussed here.

In the presented results, a polymerase effect has been added to the catalyzed reaction rate *k*_*c*_ to remedy some of the simplifying assumptions about the polymerase stated above. While we do not know about the variation in *k*_*c*_ between different cognate (or non-cognate) nucleotides, we need to add a factor that discriminates between cognate and non-cognate nucleotides in order to achieve a reasonable accuracy distribution. Without this polymerase effect, the accuracy distribution is down-shifted to values far below experimental estimates and the distribution gets truncated at 1. To investigate the accuracy distribution as it appears in the experimental range, we introduce the polymerase effect.

The structural interpretation of the polymerase effect in the catalyzed reaction is that the template-paired cognate substrate is in a better position for the phosphodiester bond formation than the non-cognate substrate. The base stacking is assumed to stay the same through the transition state. Besides shifting the entire accuracy distribution, the polymerase effect does not affect the sequence dependent variation. The results of the above mentioned simplifying assumptions about the sequence—polymerase interactions are difficult to predict. Yet, we can conclude that any effect that depend on only a couple of bases—for instance, any sequence specific effects in the transition state of phosphodiester bond formation—would have a limited effect on the wide range of outcomes in the distribution of the total accuracy, since such local effects could have only 16 possible outcomes. Future additions of this kind of effects would hence not affect the comparison between two bubbles very much, depending on only two out of at least eleven nucleotides, but could still give better predictions of the accuracy for a given motif.

## Supporting Information

S1 Fig Example from the energy landscape, including reaction rates. Note that the relation between the states might just as well have been the opposite.
(TIF)

S2 Fig Energy landscape of a proofreading cycle, comparing a correct (green) or mismatched (red) last incorporation. Each ground and transition state is labeled with the free energy of formation; ground states with state names, and transition states (marked ‡) with the names of associated reaction rates in subscript. Discrimination against the mismatch occurs when the non-cognate TEC makes a higher climb to reach the transition state when going forward, or lower when going backward, than the cognate TEC. In this example, cognate PRE is more stable than BACK due to the context sequence, but BACK, where the misincorporation is unpaired from the template, is more stable than non-cognate PRE. Therefore, both translocations between PRE and BACK, with reaction rates *k*_*1*_ and *q*_*1*_, are discriminating since they make it easier for the non-cognate complex to go backward and more difficult to go forward. The translocations to and from POST depend only on the sequence context, which affects the propensity to backtrack for both complexes and hence the accuracy, but is not discriminating since it is the same for both cognate and non-cognate complexes. The last discriminating reaction is the nucleotide dissociation at rate *q*_*3*_, where the incoming nucleotide that binds to a mismatch stabilizes the non-cognate complex less, facilitating the backward nucleotide dissociation. In this example, the new incorporation stabilizes the state non-cognate POST·NTP, so the phosphodiester bond formation is not discriminating apart from the polymerase effect, which is excluded here to show only sequence dependent energy differences.
(TIF)

S3 Fig Accuracy distributions in the *rrnC* operon for a few different parameter sets. This is a demonstration of the robustness of the results; some variables have biologically unreasonable values. The parameter sets originate from the preferred parameters, but with changes to one or two barriers by 5RT, to give a ≈150-fold difference to reaction rates. The parameter sets are: 1. the preferred parameters, as described in Methods; transit time 57 s. 2. *k*_*c*_ increased; transit time 26 s. 3. *k*_*c*_ reduced; transit time 5.5·10^7^ s. 4. *q*_*c*_ reduced; transit time 34 s. 5. *k*_*c*_ and *q*_*c*_ reduced; transit time 3.8·10^3^ s. 6. Translocation rates and association rate increased; transit time 25 s. Note that accuracy distributions 5 and 6 are identical, demonstrating that the balance between parameters determines the accuracy (but not transcription speed).
(TIF)

S1 Table The reference dataset with genome positions. The accuracy motif around each position starts 13 nucleotides in 5’ direction and ends 3 nucleotides in 3’ direction from the nucleotide in regard, on the coding strand for the transcript.
(PDF)

## References

[1] Ninio J. Connections between translation, transcription and replication error-rates. Biochimie. 1991;73: 1517–1523. 1805967 10.1016/0300-9084(91)90186-5

[2] Imashimizu M, Oshima T, Lubkowska L, Kashlev M. Direct assessment of transcription fidelity by high-resolution RNA sequencing. Nucleic acids res. 2013;41: 9090–9104. 10.1093/nar/gkt698 23925128 PMC3799451

[3] Johansson M, Zhang J, Ehrenberg M. Genetic code translation displays a linear trade-off between efficiency and accuracy of tRNA selection. Proc Natl Acad Sci USA. 2012;109: 131–136. 10.1073/pnas.1116480109 22190491 PMC3252910

[4] Gout JF, Thomas WK, Smith Z, Okamoto K, Lynch M. Large-scale detection of in vivo transcription errors. Proc Natl Acad Sci USA. 2013;110: 18584–18589. 10.1073/pnas.1309843110 24167253 PMC3832031

[5] Bouadloun F, Donner D, Kurland CG. Codon-specific missense errors in vivo. EMBO J. 1983;2: 1351–1356. 10872330 10.1002/j.1460-2075.1983.tb01591.x PMC555282

[6] Kramer EB, Farabaugh PJ. The frequency of translational misreading errors in E. coli is largely determined by tRNA competition. RNA. 2007;13: 1–10. 17095544 10.1261/rna.294907 PMC1705757

[7] Yager TD, Von Hippel PH. A thermodynamic analysis of RNA transcript elongation and termination in Escherichia coli. Biochemistry. 1991;30: 1097–1118. 1703438 10.1021/bi00218a032

[8] Bai L, Shundrovsky A, Wang MD. Sequence-dependent kinetic model for transcription elongation by RNA polymerase. J Mol Biol. 2004;344: 335–349. 15522289 10.1016/j.jmb.2004.08.107

[9] SantaLucia J. A unified view of polymer, dumbbell, and oligonucleotide DNA nearest-neighbor thermodynamics. Proc Natl Acad Sci USA. 1998;95: 1460–1465. 9465037 10.1073/pnas.95.4.1460 PMC19045

[10] Sugimoto N, Nakano S, Katoh M, Matsumura A, Nakamuta H, Ohmichi T, et al Thermodynamic parameters to predict stability of RNA/DNA hybrid duplexes. Biochemistry. 1995;34: 11211–11216. 7545436 10.1021/bi00035a029

[11] Ogle JM, Brodersen DE, Clemons WM Jr, Tarry MJ, Carter AP, Ramakrishnan V. Recognition of cognate transfer RNA by the 30S ribosomal subunit. Science. 2001;292: 897–902. 11340196 10.1126/science.1060612

[12] Ehrenberg M, Kurland CG. Costs of accuracy determined by a maximal growth rate constraint. Q Rev Biophys. 1984;17: 45–82. 6484121 10.1017/s0033583500005254

[13] Johansson M, Lovmar M, Ehrenberg M. Rate and accuracy of bacterial protein synthesis revisited. Current Opin Microbiol. 2008;11: 141–147. 10.1016/j.mib.2008.02.015 18400551

[14] Guajardo R, Sousa R. A model for the mechanism of polymerase translocation. J Mol Biol. 1997;265: 8–19. 8995520 10.1006/jmbi.1996.0707

[15] Nudler E, Mustaev A, Goldfarb A, Lukhtanov E. The RNA-DNA hybrid maintains the register of transcription by preventing backtracking of RNA polymerase. Cell. 1997;89: 33–41. 9094712 10.1016/s0092-8674(00)80180-4

[16] Korzheva N, Mustaev A, Kozlov M, Malhotra A, Nikiforov V, Goldfarb A, et al A structural model of transcription elongation. Science. 2000;289: 619–625. 10915625 10.1126/science.289.5479.619

[17] Erie DA, Hajiseyedjavadi O, Young MC, von Hippel PH. Multiple RNA polymerase conformations and GreA: control of the fidelity of transcription. Science. 1993;262: 867–873. 8235608 10.1126/science.8235608

[18] Orlova M, Newlands J, Das A, Goldfarb A, Borukhov S. Intrinsic transcript cleavage activity of RNA polymerase. Proc Natl Acad Sci USA. 1995;92: 4596–4600. 7538676 10.1073/pnas.92.10.4596 PMC41991

[19] Ehrenberg M, Blomberg C. Thermodynamic constraints on kinetic proofreading in biosynthetic pathways. Biophys J. 1980;31: 333–358. 7260292 10.1016/S0006-3495(80)85063-6 PMC1328794

[20] Bochkareva A, Yuzenkova Y, Tadigotla VR, Zenkin N. Factor-independent transcription pausing caused by recognition of the RNA-DNA hybrid sequence. EMBO J. 2012;31: 630–639. 10.1038/emboj.2011.432 22124324 PMC3273390

[21] Wang D, Bushnell DA, Huang X, Westover KD, Levitt M, Kornberg RD. Structural basis of transcription: backtracked RNA polymerase II at 3.4 angstrom resolution. Science. 2009;324: 1203–1206. 10.1126/science.1168729 19478184 PMC2718261

[22] Borukhov S, Sagitov V, Goldfarb A. Transcript cleavage factors from E. coli. Cell. 1993;72: 459–466. 8431948 10.1016/0092-8674(93)90121-6

[23] Zenkin N, Yuzenkova Y, Severinov K. Transcript-assisted transcriptional proofreading. Science. 2006;313: 518–520. 16873663 10.1126/science.1127422

[24] Bai L, Fulbright RM, Wang MD. Mechanochemical kinetics of transcription elongation. Phys Rev Lett. 2007;98: 68103–1–68103–4. 10.1103/PhysRevLett.98.068103 17358986

[25] Satpati P, Sund J, Åqvist J. Structure-based energetics of mRNA decoding on the ribosome. Biochemistry. 2014;53: 1714–1722. 10.1021/bi5000355 24564511

[26] Mellenius H, Ehrenberg M. Large DNA template dependent error variation during transcription In: Puglisi JD and Margaris MV, editors. Biophysics and Structure to Counter Threats and Challenges. Netherlands: Springer; 2013 pp 39–57.

[27] Fersht A. Structure and mechanism in protein science: A guide to enzyme catalysis and protein folding. New York: W.H. Freeman and Company; 1999 pp 103–131.

[28] Hopfield JJ. Kinetic proofreading: a new mechanism for reducing errors in biosynthetic processes requiring high specificity. Proc Natl Acad Sci USA. 1974;71: 4135–4139. 4530290 10.1073/pnas.71.10.4135 PMC434344

[29] Ninio J. Kinetic amplification of enzyme discrimination. Biochimie. 1975;57: 587–595. 1182215 10.1016/s0300-9084(75)80139-8

[30] Hopfield J, Yamane T, Yue V, Coutts S. Direct experimental evidence for kinetic proofreading in amino acylation of tRNAIle. Proc Natl Acad Sci USA. 1976;73: 1164–1168. 1063397 10.1073/pnas.73.4.1164 PMC430221

[31] Jeon CJ, Agarwal K. Fidelity of RNA polymerase II transcription controlled by elongation factor TFIIS. Proc Natl Acad Sci USA. 1996;93: 13677–13682. 8942993 10.1073/pnas.93.24.13677 PMC19388

[32] Alic N, Ayoub N, Landrieux E, Favry E, Baudouin-Cornu P, Riva M, et al Selectivity and proofreading both contribute significantly to the fidelity of RNA polymerase III transcription. Proc Natl Acad Sci USA. 2007;104: 10400–10405. 17553959 10.1073/pnas.0704116104 PMC1965525

[33] Watkins NE, Kennelly WJ, Tsay MJ, Tuin A, Swenson L, Lee H, et al Thermodynamic contributions of single internal rAdA, rCdC, rGdG and rUdT mismatches in RNA/DNA duplexes. Nucleic Acids Res. 2011;39: 1894–1902. 10.1093/nar/gkq905 21071398 PMC3061078

[34] Buckstein MH, He J, Rubin H. Characterization of nucleotide pools as a function of physiological state in Escherichia coli. J Bacteriol. 2008;190: 718–726. 17965154 10.1128/JB.01020-07 PMC2223692

[35] Blank A, Gallant JA, Burgess RR, Loeb LA. An RNA polymerase mutant with reduced accuracy of chain elongation. Biochemistry. 1986;25: 5920–5928. 3098280 10.1021/bi00368a013

[36] Itzkovitz S, Alon U. The genetic code is nearly optimal for allowing additional information within protein-coding sequences. Genome Res. 2007;17: 405–412. 17293451 10.1101/gr.5987307 PMC1832087

[37] Keseler IM, Mackie A, Peralta-Gil M, Santos-Zavaleta A, Gama-Castro S, Bonavides-Martínez C, et al EcoCyc: fusing model organism databases with systems biology. Nucleic Acids Res. 2013;41: D605–D612. 10.1093/nar/gks1027 23143106 PMC3531154

[38] Springgate CF, Loeb LA. On the fidelity of transcription by Escherichia coli ribonucleic acid polymerase. J Mol Biol. 1975;97: 577–591. 1102716 10.1016/s0022-2836(75)80060-x

[39] Larson MH, Zhou J, Kaplan CD, Palangat M, Kornberg RD, Landick R, et al Trigger loop dynamics mediate the balance between the transcriptional fidelity and speed of RNA polymerase II. Proc Natl Acad Sci USA. 2012;109: 6555–6560. 10.1073/pnas.1200939109 22493230 PMC3340090

[40] Fersht A. Structure and mechanism in protein science: A guide to enzyme catalysis and protein folding. New York: W.H. Freeman and Company; 1999 pp. 153–158.

[41] Bremer H, Dennis PP. In: Neidhardt FC, Ingraham JL, Low KB, Magasanik B, Schaechter M, Umbarger HE, editors. Escherichia coli and Salmonella typhimurium: Cellular and molecular biology. Washington D.C.: American Society for Microbiology; 1987 pp. 1527–1542.

[42] Nedialkov YA, Gong XQ, Hovde SL, Yamaguchi Y, Handa H, Geiger JH, et al NTP-driven translocation by human RNA polymerase II. J Biol Chem. 2003;278: 18303–18312. 12637520 10.1074/jbc.M301103200

[43] Miropolskaya N, Esyunina D, Klimašauskas S, Nikiforov V, Artsimovitch I, Kulbachinskiy A. Interplay between the trigger loop and the F loop during RNA polymerase catalysis. Nucleic Acids Res. 2014;42: 544–552. 10.1093/nar/gkt877 24089145 PMC3874190

[44] Sosunova E, Sosunov V, Epshtein V, Nikiforov V, Mustaev A. Control of transcriptional fidelity by active center tuning as derived from RNA polymerase endonuclease reaction. J Biol Chem. 2013;288: 6688–6703. 10.1074/jbc.M112.424002 23283976 PMC5396497

