# Supplementary figures and images for: DNA Template Dependent Accuracy Variation of Nucleotide Selection in Transcription
Source: PLoS One. 2015 Mar 23;10(3):e0119588. doi: 10.1371/journal.pone.0119588 (PMC4370716; doi:10.1371/journal.pone.0119588)

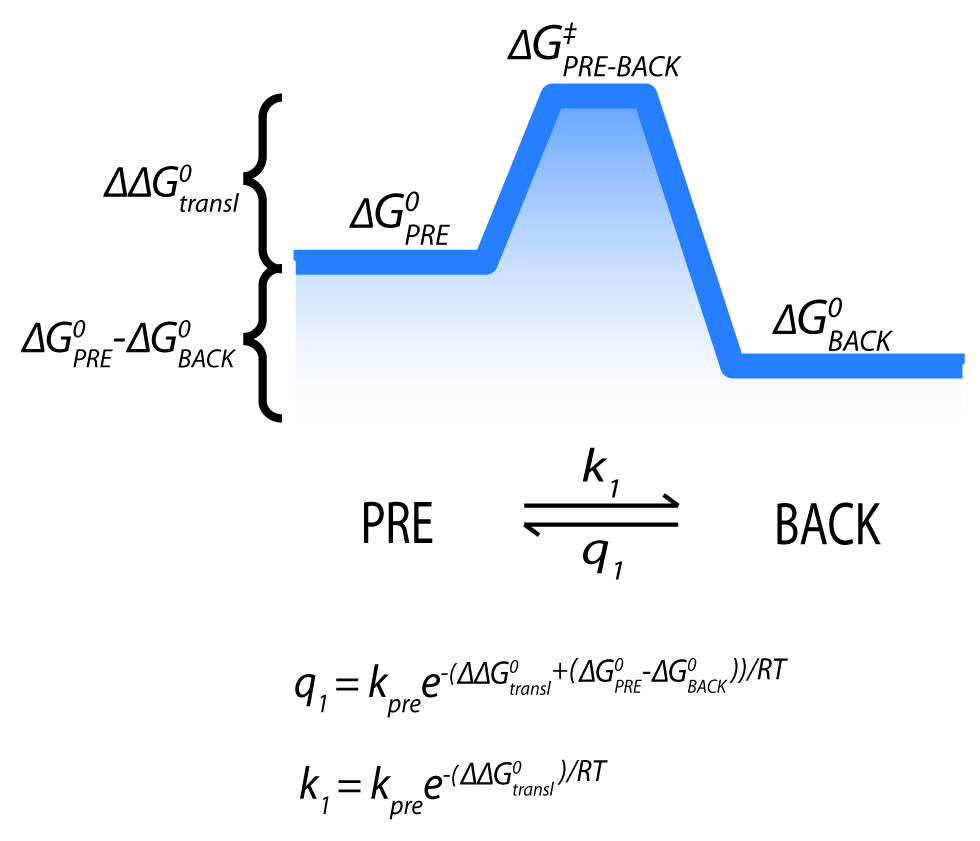

Supplement: S1 Fig — Note that the relation between the states might just as well have been the opposite. (TIF) [file pone.0119588.s001.tif]

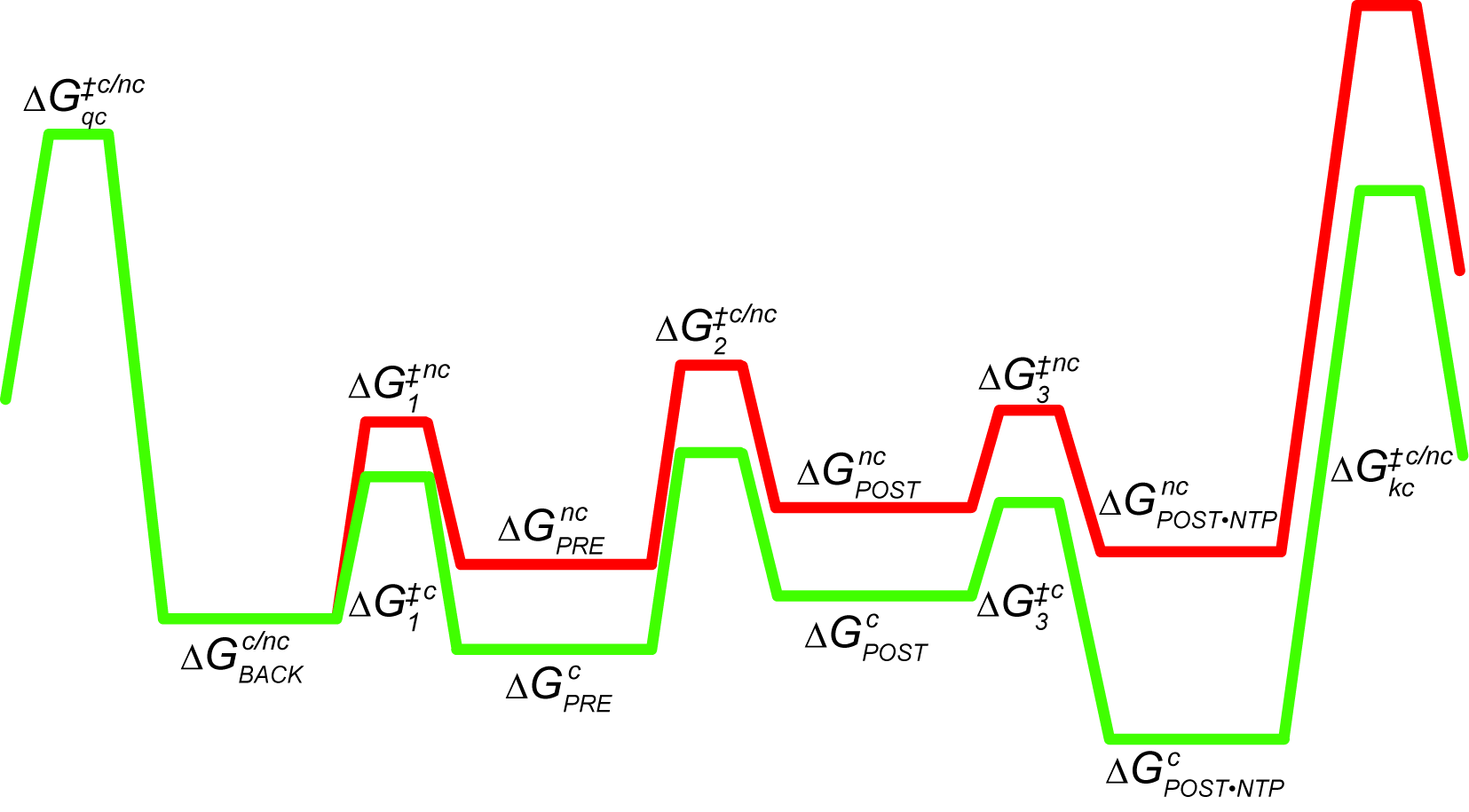

Supplement: S2 Fig — Each ground and transition state is labeled with the free energy of formation; ground states with state names, and transition states (marked ‡) with the names of associated reaction rates in subscript. Discrimination against the mismatch occurs when the non-cognate TEC makes a higher climb to reach the transition state when going forward, or lower when going backward, than the cognate TEC. In this example, cognate PRE is more stable than BACK due to the context sequence, but BACK, where the misincorporation is unpaired from the template, is more stable than non-cognate PRE. Therefore, both translocations between PRE and BACK, with reaction rates k 1 and q 1, are discriminating since they make it easier for the non-cognate complex to go backward and more difficult to go forward. The translocations to and from POST depend only on the sequence context, which affects the propensity to backtrack for both complexes and hence the accuracy, but is not discriminating since it is the same for both cognate and non-cognate complexes. The last discriminating reaction is the nucleotide dissociation at rate q 3, where the incoming nucleotide that binds to a mismatch stabilizes the non-cognate complex less, facilitating the backward nucleotide dissociation. In this example, the new incorporation stabilizes the state non-cognate POST·NTP, so the phosphodiester bond formation is not discriminating apart from the polymerase effect, which is excluded here to show only sequence dependent energy differences. (TIF) [file pone.0119588.s002.tif]

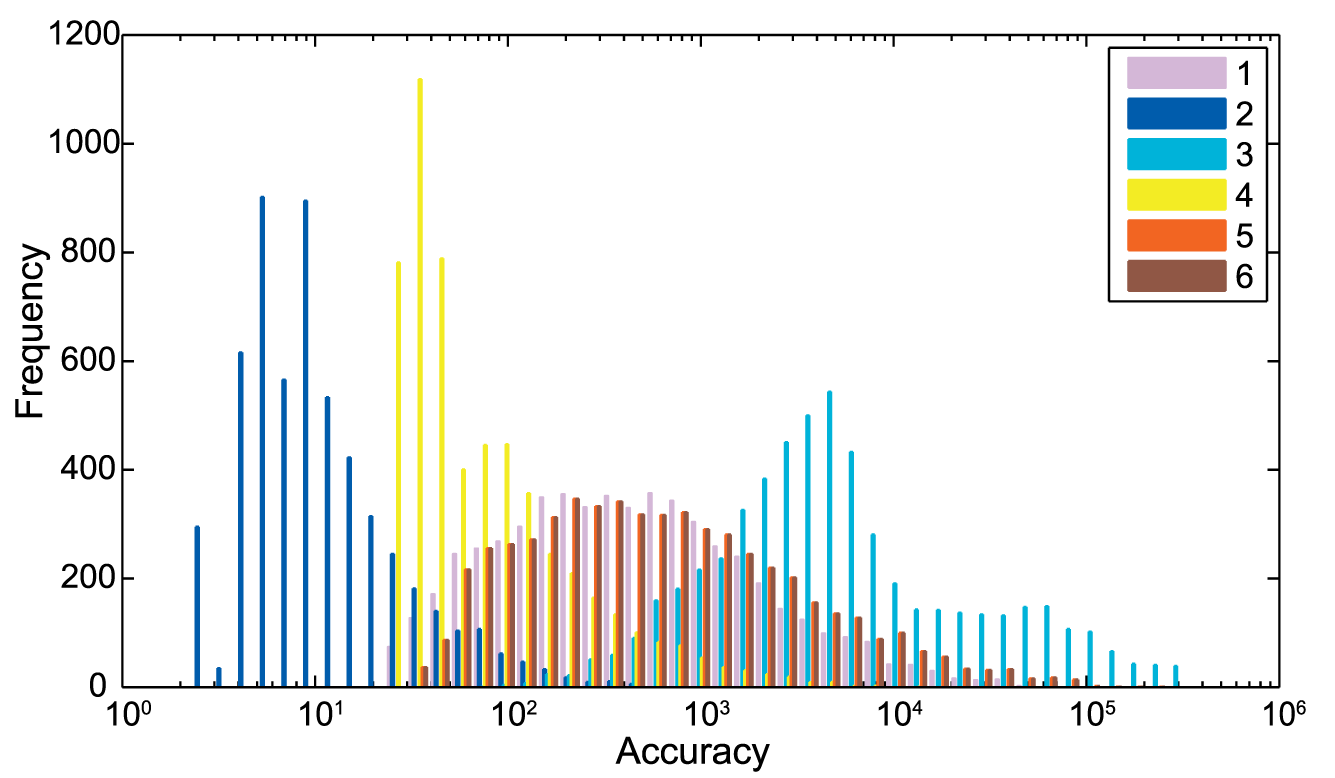

Supplement: S3 Fig — This is a demonstration of the robustness of the results; some variables have biologically unreasonable values. The parameter sets originate from the preferred parameters, but with changes to one or two barriers by 5RT, to give a ≈150-fold difference to reaction rates. The parameter sets are: 1. the preferred parameters, as described in Methods; transit time 57 s. 2. k c increased; transit time 26 s. 3. k c reduced; transit time 5.5·107 s. 4. q c reduced; transit time 34 s. 5. k c and q c reduced; transit time 3.8·103 s. 6. Translocation rates and association rate increased; transit time 25 s. Note that accuracy distributions 5 and 6 are identical, demonstrating that the balance between parameters determines the accuracy (but not transcription speed). (TIF) [file pone.0119588.s003.tif]
