# Supplementary material for: DNA Template Dependent Accuracy Variation of Nucleotide Selection in Transcription
Source: PLoS One. 2015 Mar 23;10(3):e0119588. doi: 10.1371/journal.pone.0119588 (PMC4370716; doi:10.1371/journal.pone.0119588)
Supplement: S1 Table — The accuracy motif around each position starts 13 nucleotides in 5’ direction and ends 3 nucleotides in 3’ direction from the nucleotide in regard, on the coding strand for the transcript. (PDF) [file pone.0119588.s004.pdf]

| Transcript | Chromosome | Position | Calculated accuracy |
|---|---|---|---|
| Y54F10BL.1 | III | 2261390 | 9639475.4 |
| Y54F10BL.1 | III | 2261180 | 26786.504 |
| Y54F10BL.1 | III | 2260841 | 569321.76 |
| Y54F10BL.1 | III | 2260814 | 784338.65 |
| Y54F10BL.1 | III | 2260701 | 9844.9463 |
| Y54F10BL.1 | III | 2260678 | 67020.45 |
| Y54F10BL.1 | III | 2260515 | 1630.4565 |
| Y54F10BL.1 | III | 2260483 | 3427889 |
| Y54F10BL.1 | III | 2260352 | 4980.1491 |
| Y54F10BL.1 | III | 2260332 | 9319109.6 |
| Y54F10BL.1 | III | 2260176 | 33953.918 |
| Y54F10BL.1 | III | 2260124 | 13890.441 |
| Y54F10BL.1 | III | 2260069 | 151167.39 |
| Y54F10BL.1 | III | 2260009 | 128915.06 |
| Y54F10BL.1 | III | 2259978 | 59264.257 |
| Y54F10BL.1 | III | 2259926 | 353830.28 |
| Y54F10BL.1 | III | 2259904 | 18238.898 |
| F07E5.6 | II | 2063799 | 1285240 |
| F07E5.6 | II | 2063785 | 29418128 |
| F07E5.6 | II | 2063764 | 1403464.2 |
| F07E5.6 | II | 2063714 | 78398.245 |
| F07E5.6 | II | 2063636 | 3765200.4 |
| F07E5.6 | II | 2063618 | 2488.8905 |
| F07E5.6 | II | 2063573 | 5222.8143 |
| F07E5.6 | II | 2063560 | 24218.619 |
| F07E5.6 | II | 2063521 | 88194.739 |
| F07E5.6 | II | 2063515 | 265187.02 |
| F07E5.6 | II | 2063498 | 103216.42 |
| F07E5.6 | II | 2063289 | 8782040.5 |
| F07E5.6 | II | 2063248 | 2102332.4 |
| F07E5.6 | II | 2063216 | 7673.2696 |
| F07E5.6 | II | 2061800 | 1339559.4 |
| F07E5.6 | II | 2061776 | 4745880.6 |
| F07E5.6 | II | 2061749 | 6963.8846 |
| F07E5.6 | II | 2061420 | 1510766.3 |
| F07E5.6 | II | 2061345 | 7355.7675 |
| F07E5.6 | II | 2060971 | 46770.308 |
| F07E5.6 | II | 2060921 | 2222346.5 |
| F07E5.6 | II | 2060899 | 16845.793 |
| F07E5.6 | II | 2060888 | 41914.75 |
| F07E5.6 | II | 2060635 | 49020.052 |
| F07E5.6 | II | 2060633 | 87417.793 |
| F07E5.6 | II | 2060558 | 872.32848 |
| F07E5.6 | II | 2060550 | 643.65828 |

| | | | |
|---|---|---|---|
| F07E5.6 | II | 2060527 | 1653552.1 |
| F07E5.6 | II | 2060498 | 138196.18 |
| F07E5.6 | II | 2060436 | 202560.92 |
| F07E5.6 | II | 2059898 | 2070666.3 |
| F07E5.6 | II | 2059880 | 6993.4082 |
| F07E5.6 | II | 2059813 | 545579.57 |
| F07E5.6 | II | 2059799 | 131449.36 |
| W03F11.3 | I | 2234770 | 4146.2945 |
| W03F11.3 | I | 2234338 | 2530457.9 |
| W03F11.3 | I | 2234335 | 132566.31 |
| W03F11.3 | I | 2234322 | 54631481 |
| W03F11.3 | I | 2234224 | 278739.58 |
| W03F11.3 | I | 2234179 | 11733.066 |
| W03F11.3 | I | 2234147 | 40786.156 |
| W03F11.3 | I | 2233851 | 55459.745 |
| M70.3a | IV | 2242256 | 2480.3383 |
| M70.3a | IV | 2240694 | 215625.02 |
| M70.3a | IV | 2240579 | 678634.02 |
| M70.3a | IV | 2240527 | 107648.17 |
| M70.3a | IV | 2240501 | 39697.501 |
| M70.3a | IV | 2240473 | 51842.262 |
| M70.3a | IV | 2240453 | 150331.89 |
| M70.3a | IV | 2240413 | 2422.7583 |
| M70.3a | IV | 2240238 | 932440.56 |
| M70.3a | IV | 2240064 | 6740.5181 |
| M70.3a | IV | 2239995 | 498186.5 |
| M70.3a | IV | 2239977 | 82085.901 |
| M70.3a | IV | 2239948 | 6676.938 |
| M70.3a | IV | 2239947 | 18655.006 |
| M70.3a | IV | 2239946 | 290917.74 |
| M70.3a | IV | 2239930 | 28718.043 |
| M70.3a | IV | 2239917 | 83853.127 |
| M70.3a | IV | 2239706 | 298762.8 |
| M70.3a | IV | 2239656 | 5274.6325 |
| M70.3a | IV | 2239620 | 6831.6788 |
| M70.3a | IV | 2239562 | 22687.313 |
| M70.3a | IV | 2239561 | 2708.3859 |
| M70.3a | IV | 2239526 | 171240.36 |
| M70.3a | IV | 2239501 | 301046.17 |
| M70.3a | IV | 2239433 | 5266787.6 |
| M70.3a | IV | 2239417 | 241281.11 |
| M70.3a | IV | 2239367 | 10959.582 |
| M70.3a | IV | 2239352 | 11351.738 |
| M70.3a | IV | 2239075 | 68116.71 |
| M70.3a | IV | 2239071 | 591161.1 |
| M70.3a | IV | 2238987 | 9286.8293 |
| M70.3a | IV | 2238743 | 3579685.7 |
| M70.3a | IV | 2238732 | 952520.82 |
| M70.3a | IV | 2238652 | 14673.13 |

| | | | |
|---|---|---|---|
| M70.3a | IV | 2238553 | 68384.778 |
| M70.3a | IV | 2238536 | 24132.728 |
| M70.3a | IV | 2238456 | 187581.73 |
| M70.3a | IV | 2238220 | 45993.138 |
| M70.3a | IV | 2237757 | 30888.432 |
| M70.3a | IV | 2237721 | 1464.2741 |
| Y40B10A.9 | V | 2072844 | 1298328.6 |
| Y40B10A.9 | V | 2072829 | 1345.7665 |
| Y40B10A.9 | V | 2072822 | 40349.285 |
| Y40B10A.9 | V | 2072821 | 114189.7 |
| Y40B10A.9 | V | 2072770 | 13958.106 |
| Y40B10A.9 | V | 2072761 | 2206.6059 |
| Y40B10A.9 | V | 2071615 | 102347.24 |
| Y40B10A.9 | V | 2071575 | 543096.7 |
| Y40B10A.9 | V | 2071526 | 12959800 |
| Y40B10A.9 | V | 2071510 | 982.60274 |
| Y40B10A.9 | V | 2071507 | 134372.32 |
| Y40B10A.9 | V | 2070299 | 2022675.6 |
| Y40B10A.9 | V | 2070294 | 36225.778 |
| Y40B10A.9 | V | 2070283 | 25370.859 |
| Y40B10A.9 | V | 2070272 | 5011.4767 |
| Y40B10A.9 | V | 2070204 | 5826.4053 |
| Y40B10A.9 | V | 2070147 | 2997603.2 |
| Y40B10A.9 | V | 2070124 | 448906.58 |
| Y40B10A.9 | V | 2069990 | 7991.7673 |
| Y40B10A.9 | V | 2069986 | 4342.1846 |
| Y40B10A.9 | V | 2069684 | 2015.4888 |
| Y40B10A.9 | V | 2069369 | 77823274 |
| Y40B10A.9 | V | 2069268 | 76088.07 |
| Y40B10A.9 | V | 2069216 | 519386.37 |
| F58D5.2b.1 | I | 12068600 | 1155089.2 |
| F58D5.2b.1 | I | 12068527 | 878559.56 |
| F58D5.2b.1 | I | 12068516 | 35215.341 |
| F58D5.2b.1 | I | 12068136 | 867.14664 |
| F58D5.2b.1 | I | 12068096 | 1.23E+09 |
| F58D5.2b.1 | I | 12068074 | 14766.524 |
| F58D5.2b.1 | I | 12068070 | 1762.3665 |
| F58D5.2b.1 | I | 12067983 | 84254.35 |
| F58D5.2b.1 | I | 12066266 | 56979.156 |
| F58D5.2b.1 | I | 12066264 | 7287.8459 |
| F58D5.2b.1 | I | 12066139 | 11349.47 |
| F58D5.2b.1 | I | 12066082 | 789351.08 |
| F58D5.2b.1 | I | 12066004 | 6136.9216 |
| F58D5.2b.1 | I | 12065942 | 1513915.8 |
| T12G3.2a.1 | IV | 12042765 | 322273.55 |
| T12G3.2a.1 | IV | 12042736 | 6416.0794 |
| T12G3.2a.1 | IV | 12042724 | 7015.1705 |
| T12G3.2a.1 | IV | 12042689 | 100549.28 |
| T12G3.2a.1 | IV | 12042685 | 68107.372 |

| | | | |
|---|---|---|---|
| T12G3.2a.1 | IV | 12042585 | 249440.27 |
| T12G3.2a.1 | IV | 12042534 | 7636.1466 |
| T12G3.2a.1 | IV | 12042509 | 2848821 |
| T12G3.2a.1 | IV | 12042350 | 2707.5884 |
| T12G3.2a.1 | IV | 12042347 | 94882.496 |
| T12G3.2a.1 | IV | 12042298 | 10497.098 |
| T12G3.2a.1 | IV | 12042296 | 2745012.4 |
| T12G3.2a.1 | IV | 12042141 | 8269.5527 |
| T12G3.2a.1 | IV | 12042087 | 725.33403 |
| T12G3.2a.1 | IV | 12041845 | 7013.0697 |
| T12G3.2a.1 | IV | 12041844 | 52872.701 |
| T12G3.2a.1 | IV | 12041839 | 219379.45 |
| T12G3.2a.1 | IV | 12041785 | 2879.7984 |
| T12G3.2a.1 | IV | 12041725 | 53774.272 |
| T12G3.2a.1 | IV | 12041631 | 4605315.4 |
| T12G3.2a.1 | IV | 12041599 | 177942.52 |
| T12G3.2a.1 | IV | 12041547 | 37750.323 |
| T12G3.2a.1 | IV | 12041493 | 51879.769 |
| T12G3.2a.1 | IV | 12041485 | 129164.78 |
| T12G3.2a.1 | IV | 12041474 | 25937.007 |
| T12G3.2a.1 | IV | 12041447 | 13348.856 |
| T12G3.2a.1 | IV | 12041432 | 169476.25 |
| T12G3.2a.1 | IV | 12041367 | 975376.64 |
| T12G3.2a.1 | IV | 12041231 | 2097731.5 |
| T12G3.2a.1 | IV | 12041167 | 125341.25 |
| T12G3.2a.1 | IV | 12041155 | 5236.2608 |
| T12G3.2a.1 | IV | 12041123 | 9932.6555 |
| T12G3.2a.1 | IV | 12040822 | 212095.21 |
| T12G3.2a.1 | IV | 12040816 | 2934.9725 |
| T12G3.2a.1 | IV | 12040806 | 24103.341 |
| T12G3.2a.1 | IV | 12040409 | 3766168.1 |
| T12G3.2a.1 | IV | 12040360 | 4744.9405 |
| T12G3.2a.1 | IV | 12040355 | 155975.34 |
| T12G3.2a.1 | IV | 12040189 | 24860.607 |
| T12G3.2a.1 | IV | 12040159 | 5070.1652 |
| T12G3.2a.1 | IV | 12040158 | 1538172.2 |
| T12G3.2a.1 | IV | 12039702 | 47266.175 |
| T12G3.2a.1 | IV | 12039680 | 168602.3 |
| T12G3.2a.1 | IV | 12039614 | 154852.03 |
| T12G3.2a.1 | IV | 12039611 | 17071.563 |
| T12G3.2a.1 | IV | 12039599 | 35486.341 |
| T12G3.2a.1 | IV | 12039518 | 7784.9438 |
| T12G3.2a.1 | IV | 12039432 | 523079.95 |
| T12G3.2a.1 | IV | 12039428 | 205173.38 |
| T12G3.2a.1 | IV | 12039305 | 2713999.2 |
| T12G3.2a.1 | IV | 12039245 | 385421.7 |
| T12G3.2a.1 | IV | 12039185 | 509555.4 |
| T12G3.2a.1 | IV | 12039173 | 66362.43 |
| T12G3.2a.1 | IV | 12039172 | 13440.359 |

| | | | |
|---|---|---|---|
| T12G3.2a.1 | IV | 12039074 | 6877.3366 |
| T12G3.2a.1 | IV | 12039031 | 231710.54 |
| T12G3.2a.1 | IV | 12039028 | 1945.4259 |
| T12G3.2a.1 | IV | 12039025 | 598416.94 |
| T12G3.2a.1 | IV | 12039023 | 111003.93 |
| R07B7.3a | V | 12065329 | 2765435.8 |
| R07B7.3a | V | 12065327 | 42574.272 |
| R07B7.3a | V | 12065306 | 4698904.2 |
| R07B7.3a | V | 12065091 | 12948.115 |
| R07B7.3a | V | 12065057 | 6014119.5 |
| R07B7.3a | V | 12065041 | 214232.71 |
| R07B7.3a | V | 12064969 | 6254302.1 |
| R07B7.3a | V | 12064274 | 90937.557 |
| R07B7.3a | V | 12064254 | 4416.1125 |
| R07B7.3a | V | 12064101 | 11655.386 |
| R07B7.3a | V | 12064076 | 4598.9198 |
| R07B7.3a | V | 12063995 | 3043.4789 |
| R07B7.3a | V | 12063894 | 1567816.7 |
| R07B7.3a | V | 12063840 | 2494.413 |
| R07B7.3a | V | 12063585 | 468394.79 |
| R07B7.3a | V | 12063554 | 25468.044 |
| Y17G7B.23a | II | 11999400 | 157990.29 |
| Y17G7B.23a | II | 11999410 | 6844.8902 |
| Y17G7B.23a | II | 11999569 | 39813.992 |
| Y17G7B.23a | II | 12000385 | 43440.207 |
| Y17G7B.23a | II | 12000537 | 1060479.6 |
| Y17G7B.23a | II | 12000715 | 162162.2 |
| Y17G7B.23a | II | 12000723 | 8286.7282 |
| Y17G7B.23a | II | 12000786 | 1360319.4 |
| Y17G7B.23a | II | 12000828 | 433778.12 |
| Y17G7B.23a | II | 12000844 | 291342.78 |
| Y17G7B.23a | II | 12001340 | 1404.0334 |
| Y75B8A.23 | III | 12250574 | 4505.9243 |
| Y75B8A.23 | III | 12250784 | 3559.2875 |
| Y75B8A.23 | III | 12250858 | 2737.358 |
| ZK512.1 | III | 9115124 | 31097691 |
| ZK512.1 | III | 9115161 | 1744.5905 |
| ZK512.1 | III | 9115548 | 1225868.2 |
| ZK512.1 | III | 9116405 | 171072.72 |
| ZK512.1 | III | 9116899 | 30056526 |
| ZK512.1 | III | 9116901 | 5119.9511 |
| ZK512.1 | III | 9117009 | 222574.01 |
| ZK512.1 | III | 9117058 | 11215.308 |
| ZK512.1 | III | 9117176 | 3421.3466 |
| ZK858.4 | I | 9132613 | 224048.29 |
| ZK858.4 | I | 9132672 | 169975.75 |
| ZK858.4 | I | 9132696 | 80317.643 |
| ZK858.4 | I | 9132721 | 74324.023 |
| ZK858.4 | I | 9132755 | 12146.823 |

| | | | |
|---|---|---|---|
| ZK858.4 | I | 9132838 | 2666.6371 |
| ZK858.4 | I | 9132844 | 18009.999 |
| ZK858.4 | I | 9132860 | 1427171.5 |
| ZK858.4 | I | 9133014 | 221110.25 |
| ZK858.4 | I | 9133899 | 263170.39 |
| ZK858.4 | I | 9134202 | 127273.9 |
| ZK858.4 | I | 9134256 | 3909.7766 |
| ZK858.4 | I | 9134287 | 1520.2284 |
| ZK858.4 | I | 9134404 | 1444869.2 |
| ZK858.4 | I | 9134431 | 936469.69 |
| ZK858.4 | I | 9134980 | 1339.6574 |
| ZK858.4 | I | 9135029 | 2851.7019 |
| ZK858.4 | I | 9135030 | 10449.235 |
| ZK858.4 | I | 9135034 | 24639.582 |
| ZK858.4 | I | 9135141 | 273191.59 |
| ZK858.4 | I | 9135163 | 7058.3218 |
| ZK858.4 | I | 9135196 | 1199.2748 |
| ZK858.4 | I | 9135231 | 6098.0904 |
| ZK858.4 | I | 9135235 | 3167930.7 |
| ZK858.4 | I | 9135259 | 162719.38 |
| ZK858.4 | I | 9135423 | 3271.432 |
| ZK858.4 | I | 9135430 | 117468.98 |
| ZK858.4 | I | 9135443 | 25470.635 |
| ZK858.4 | I | 9135496 | 5473.8091 |
| ZK858.4 | I | 9135710 | 49222.217 |
| ZK858.4 | I | 9135732 | 18615.573 |
| C46C2.6a | IV | 9213092 | 79528.668 |
| C46C2.6a | IV | 9213226 | 1866354.7 |
| C46C2.6a | IV | 9213258 | 2190308.3 |
| C46C2.6a | IV | 9213596 | 78659.033 |
| C46C2.6a | IV | 9213611 | 46457.331 |
| C46C2.6a | IV | 9213621 | 4106694.5 |
| C46C2.6a | IV | 9213711 | 106896.4 |
| C46C2.6a | IV | 9214161 | 1342046.2 |
| C46C2.6a | IV | 9214268 | 19917.291 |
| C46C2.6a | IV | 9214293 | 47484.802 |
| C46C2.6a | IV | 9214311 | 80131.159 |
| C46C2.6a | IV | 9214313 | 3305.1341 |
| C46C2.6a | IV | 9214337 | 5855.6214 |
| C46C2.6a | IV | 9214370 | 75830.493 |
| T27E4.1 | V | 9092641 | 2657625.1 |
| T27E4.1 | V | 9092674 | 2569.3776 |
| T27E4.1 | V | 9093024 | 663044.81 |
| T27E4.1 | V | 9093035 | 824642.01 |
| T27E4.1 | V | 9093052 | 6661154 |
| T27E4.1 | V | 9093312 | 202042.12 |
| T27E4.1 | V | 9093323 | 205302.45 |
| T27E4.1 | V | 9093619 | 33115.923 |
| T27E4.1 | V | 9093627 | 517987 |

| | | | |
|---|---|---|---|
| T27E4.1 | V | 9093693 | 48224.374 |
| T27E4.1 | V | 9093715 | 226162.18 |
| T27E4.1 | V | 9093894 | 3121928.6 |
| T27E4.1 | V | 9094041 | 16404844 |
| T27E4.1 | V | 9094124 | 2395270.6 |
| T27E4.1 | V | 9094221 | 83312.689 |
| T27E4.1 | V | 9094313 | 85816.901 |
| T27E4.1 | V | 9094325 | 136403.81 |
| T23F4.2 | II | 1163651 | 77387.844 |
| T23F4.2 | II | 1163974 | 883.21746 |
| T23F4.2 | II | 1164237 | 33320590 |
| T23F4.2 | II | 1164306 | 20207.659 |
| T23F4.2 | II | 1164497 | 7495.0989 |
| T23F4.2 | II | 1164499 | 39552.348 |
| T23F4.2 | II | 1164525 | 17034.385 |
| T23F4.2 | II | 1165105 | 1941.4751 |
| T23F4.2 | II | 1165140 | 5259459.8 |
| T23F4.2 | II | 1165164 | 4533.1714 |
| T23F4.2 | II | 1165213 | 75067.302 |
| T23F4.2 | II | 1165243 | 64520.076 |
| T23F4.2 | II | 1165282 | 125924.13 |
| T23F4.2 | II | 1165319 | 3570.9808 |
| T23F4.2 | II | 1165326 | 4491.2966 |
| T23F4.2 | II | 1165370 | 52774.107 |
| T23F4.2 | II | 1165407 | 18445.359 |
| T23F4.2 | II | 1165469 | 11805.689 |
| C46E10.6 | II | 3709604 | 1550026.6 |
| C46E10.6 | II | 3709609 | 100776.97 |
| C46E10.6 | II | 3709685 | 11220.599 |
| C46E10.6 | II | 3709706 | 14843.278 |
| C46E10.6 | II | 3709711 | 323776.7 |
| C46E10.6 | II | 3709838 | 5663.722 |
| C46E10.6 | II | 3709898 | 483516.2 |
| C46E10.6 | II | 3709923 | 43406.302 |
| C46E10.6 | II | 3710146 | 7012.2257 |
| C46E10.6 | II | 3710337 | 8339602.2 |
| C46E10.6 | II | 3710362 | 19292.75 |
| C46E10.6 | II | 3710383 | 20559029 |
| C46E10.6 | II | 3710398 | 1633937.2 |
| C46E10.6 | II | 3710416 | 82557.777 |
| C46E10.6 | II | 3710467 | 185837 |
| C46E10.6 | II | 3710528 | 85723.366 |
| C46E10.6 | II | 3710529 | 128513765 |
| C46E10.6 | II | 3710549 | 20065.613 |
| C46E10.6 | II | 3710571 | 454668.66 |
| C46E10.6 | II | 3710661 | 295931 |
| C46E10.6 | II | 3710699 | 846749.81 |
| C46E10.6 | II | 3710709 | 5855.1469 |
| C46E10.6 | II | 3710738 | 1002349.4 |

| | | | |
|---|---|---|---|
| C46E10.6 | II | 3710766 | 1249.5909 |
| Y71A12B.10 | I | 13957206 | 18299.455 |
| Y71A12B.10 | I | 13957216 | 2395.076 |
| Y71A12B.10 | I | 13957235 | 183607.86 |
| Y71A12B.10 | I | 13957322 | 5132489.7 |
| Y71A12B.10 | I | 13957447 | 430740.94 |
| Y71A12B.10 | I | 13957505 | 107435.07 |
| Y71A12B.10 | I | 13957566 | 56790.009 |
| Y71A12B.10 | I | 13957623 | 18510.577 |
| Y71A12B.10 | I | 13957624 | 3685.7733 |
| Y71A12B.10 | I | 13957694 | 3379668.7 |
| Y71A12B.10 | I | 13958885 | 9623.523 |
| Y71A12B.10 | I | 13958938 | 4541.6366 |
| Y71A12B.10 | I | 13959038 | 15278.797 |
| Y71A12B.10 | I | 13959053 | 31173.571 |
| Y71A12B.10 | I | 13959104 | 44685.918 |
| Y71A12B.10 | I | 13959106 | 5155.4501 |
| Y71A12B.10 | I | 13959140 | 204935.88 |
| Y71A12B.10 | I | 13959280 | 27966.558 |
| Y71A12B.10 | I | 13959285 | 20867.305 |
| Y71A12B.10 | I | 13959504 | 1251.2233 |
| Y71A12B.10 | I | 13959522 | 53390.164 |
| Y71A12B.10 | I | 13959550 | 5282.2893 |
| Y71A12B.10 | I | 13959969 | 1928116.9 |
| Y71A12B.10 | I | 13959996 | 37677.38 |
| Y71A12B.10 | I | 13960025 | 4246.3181 |
| Y71A12B.10 | I | 13960425 | 2622137.8 |
| ZK112.1 | III | 7758680 | 52886.236 |
| ZK112.1 | III | 7758682 | 3460.2234 |
| ZK112.1 | III | 7758712 | 3576.0669 |
| ZK112.1 | III | 7758714 | 744.46043 |
| ZK112.1 | III | 7758717 | 4189417.7 |
| ZK112.1 | III | 7759068 | 1468502.4 |
| ZK112.1 | III | 7759135 | 3218.3382 |
| ZK112.1 | III | 7759135 | 3218.3382 |
| ZK112.1 | III | 7759333 | 12961.082 |
| ZK112.1 | III | 7759500 | 9249.5207 |
| ZK112.1 | III | 7759572 | 401271.7 |
| ZK112.1 | III | 7759577 | 7079126.6 |
| ZK112.1 | III | 7759614 | 89272.517 |
| ZK112.1 | III | 7759837 | 134262.76 |
| ZK112.1 | III | 7759860 | 352879.85 |
| ZK112.1 | III | 7759877 | 8884.1653 |
| ZK112.1 | III | 7759953 | 32512.92 |
| ZK112.1 | III | 7759965 | 16502.266 |
| ZK112.1 | III | 7759969 | 11081.042 |
| ZK112.1 | III | 7759972 | 1451843.2 |
| ZK112.1 | III | 7760246 | 7614.6185 |
| ZK112.1 | III | 7760257 | 235743.12 |

| | | | |
|---|---|---|---|
| ZK112.1 | III | 7760341 | 19692.015 |
| ZK112.1 | III | 7760370 | 3298.9678 |
| ZK112.1 | III | 7760570 | 2754852.8 |
| ZK112.1 | III | 7760610 | 112089.82 |
| ZK112.1 | III | 7760875 | 5683.6566 |
| ZK112.1 | III | 7761011 | 164253.31 |
| ZK112.1 | III | 7761030 | 313701.69 |
| ZK112.1 | III | 7761071 | 174430.59 |
| ZK112.1 | III | 7761108 | 1330008 |
| ZK112.1 | III | 7761137 | 30466.862 |
| ZK112.1 | III | 7761148 | 50811.176 |
| ZK112.1 | III | 7761178 | 1390881.9 |
| ZK112.1 | III | 7761185 | 11373.008 |
| ZK1248.14 | II | 5835985 | 12315.538 |
| ZK1248.14 | II | 5835976 | 9157.1146 |
| ZK1248.14 | II | 5835935 | 97050.211 |
| ZK1248.14 | II | 5835780 | 1424485.4 |
| ZK1248.14 | II | 5835755 | 191960.92 |
| ZK1248.14 | II | 5835748 | 1196044.7 |
| ZK1248.14 | II | 5835744 | 746838.48 |
| ZK1248.14 | II | 5835686 | 102577.82 |
| ZK1248.14 | II | 5835684 | 3545746.8 |
| ZK1248.14 | II | 5835549 | 445001.39 |
| ZK1248.14 | II | 5835517 | 179707997 |
| ZK1248.14 | II | 5835512 | 94472.388 |
| ZK1248.14 | II | 5835481 | 883614.35 |
| ZK1248.14 | II | 5835477 | 61682 |
| ZK1248.14 | II | 5835435 | 169144.26 |
| ZK1248.14 | II | 5835408 | 13801.011 |
| ZK1248.14 | II | 5835380 | 54280.134 |
| ZK1248.14 | II | 5835320 | 180390.49 |
| ZK1248.14 | II | 5835203 | 131200358 |
| ZK1248.14 | II | 5835160 | 58845.451 |
| ZK1248.14 | II | 5835103 | 603078.4 |
| ZK1248.14 | II | 5835057 | 257292.97 |
| ZK1248.14 | II | 5834870 | 84930.901 |
| ZK1248.14 | II | 5834863 | 8687.4145 |
| ZK1248.14 | II | 5834784 | 46820.073 |
| ZK1248.14 | II | 5834606 | 2645155.9 |
| ZK1248.14 | II | 5834440 | 211476.61 |
| ZK1248.14 | II | 5834429 | 2388267.3 |
| ZK1248.14 | II | 5834372 | 157772.25 |
| ZK1248.14 | II | 5834294 | 49247.177 |
| ZK1248.14 | II | 5834220 | 6574166.5 |
| ZK1248.14 | II | 5834150 | 2798953.2 |
| ZK1248.14 | II | 5834149 | 19828.651 |
| ZK1248.14 | II | 5834094 | 23479.614 |
| ZK1248.14 | II | 5834094 | 23479.614 |
| ZK1248.14 | II | 5834035 | 34605.143 |

| | | | |
|---|---|---|---|
| ZK1248.14 | II | 5833998 | 29659758 |
| ZK1248.14 | II | 5833966 | 155684.2 |
| ZK1248.14 | II | 5833901 | 95637.169 |
| ZK1248.14 | II | 5833831 | 4853.8528 |
| ZK1248.14 | II | 5833762 | 35360.351 |
| ZK1248.14 | II | 5833727 | 20377.366 |
| ZK1248.14 | II | 5833659 | 1292943.1 |
| ZK1248.14 | II | 5833355 | 2829.4639 |
| C31H1.1 | IV | 5797425 | 5432.7503 |
| C31H1.1 | IV | 5797485 | 17575.298 |
| C31H1.1 | IV | 5797677 | 1309.7494 |
| C31H1.1 | IV | 5797878 | 4471.6147 |
| C31H1.1 | IV | 5797880 | 68087.089 |
| C31H1.1 | IV | 5797987 | 156392.13 |
| C31H1.1 | IV | 5798057 | 201199.2 |
| C31H1.1 | IV | 5798105 | 272363.31 |
| C31H1.1 | IV | 5798109 | 690828.07 |
| C31H1.1 | IV | 5798210 | 10149.997 |
| C31H1.1 | IV | 5798496 | 34221087 |
| C31H1.1 | IV | 5798562 | 2499.0861 |
| C31H1.1 | IV | 5798590 | 1269924 |
| C31H1.1 | IV | 5798591 | 28972.086 |
| C31H1.1 | IV | 5798607 | 1919.7933 |
| C31H1.1 | IV | 5798626 | 901770.23 |
| C31H1.1 | IV | 5798650 | 268563.62 |
| C31H1.1 | IV | 5798772 | 24850791 |
| C31H1.1 | IV | 5798872 | 3861353.1 |
| C31H1.1 | IV | 5798948 | 572713.54 |
| C31H1.1 | IV | 5798970 | 513246.95 |
| C31H1.1 | IV | 5798995 | 2575.891 |
| C31H1.1 | IV | 5799015 | 20667564 |
| C31H1.1 | IV | 5799108 | 6932.8097 |
| C31H1.1 | IV | 5799188 | 56604.336 |
| C31H1.1 | IV | 5799189 | 3700358.8 |
| C31H1.1 | IV | 5799249 | 9969.0858 |
| C31H1.1 | IV | 5799288 | 6339031.7 |
| C31H1.1 | IV | 5799300 | 124899.81 |
| C31H1.1 | IV | 5799501 | 20457096 |
| C31H1.1 | IV | 5799555 | 222304.07 |
| C31H1.1 | IV | 5799834 | 1613182.6 |
| C31H1.1 | IV | 5799971 | 3584222.1 |
| C31H1.1 | IV | 5800079 | 9572.7783 |
| C31H1.1 | IV | 5800157 | 13942.725 |
